# Age-Specific Gene Expression Signatures for Breast Tumors and Cross-Species Conserved Potential Cancer Progression Markers in Young Women

**DOI:** 10.1371/journal.pone.0063204

**Published:** 2013-05-21

**Authors:** Dilek Colak, Asmaa Nofal, AlBandary AlBakheet, Maimoona Nirmal, Hatim Jeprel, Abdelmoneim Eldali, Taher AL-Tweigeri, Asma Tulbah, Dahish Ajarim, Osama Al Malik, Mehmet S. Inan, Namik Kaya, Ben H. Park, Suad M. Bin Amer

**Affiliations:** 1 Department of Biostatistics, Epidemiology and Scientific Computing, King Faisal Specialist Hospital and Research Centre, Riyadh, Saudi Arabia; 2 Department of Molecular Oncology, King Faisal Specialist Hospital and Research Centre, Riyadh, Saudi Arabia; 3 Department of Genetics, King Faisal Specialist Hospital and Research Centre, Riyadh, Saudi Arabia; 4 Department of Oncology, King Faisal Specialist Hospital and Research Centre, Riyadh, Saudi Arabia; 5 Deparment of Pathology and Laboratory Medicine, King Faisal Specialist Hospital and Research Centre, Riyadh, Saudi Arabia; 6 Department of Surgery, King Faisal Specialist Hospital and Research Centre, Riyadh, Saudi Arabia; 7 The Sidney Kimmel Comprehensive Cancer Center at Johns Hopkins, Breast Cancer Research Program, Department of Oncology, Johns Hopkins University School of Medicine, Baltimore, Maryland, United States of America; Harvard School of Public Health, United States of America

## Abstract

Breast cancer in young women is more aggressive with a poorer prognosis and overall survival compared to older women diagnosed with the disease. Despite recent research, the underlying biology and molecular alterations that drive the aggressive nature of breast tumors associated with breast cancer in young women have yet to be elucidated. In this study, we performed transcriptomic profile and network analyses of breast tumors arising in Middle Eastern women to identify age-specific gene signatures. Moreover, we studied molecular alterations associated with cancer progression in young women using cross-species comparative genomics approach coupled with copy number alterations (CNA) associated with breast cancers from independent studies. We identified 63 genes specific to tumors in young women that showed alterations distinct from two age cohorts of older women. The network analyses revealed potential critical regulatory roles for Myc, PI3K/Akt, NF-κB, and IL-1 in disease characteristics of breast tumors arising in young women. Cross-species comparative genomics analysis of progression from pre-invasive ductal carcinoma *in situ* (DCIS) to invasive ductal carcinoma (IDC) revealed 16 genes with concomitant genomic alterations, *CCNB2, UBE2C, TOP2A*, *CEP55*, TPX2, *BIRC5, KIAA0101*, *SHCBP1*, *UBE2T*, *PTTG1*, *NUSAP1*, *DEPDC1*, *HELLS*, *CCNB1*, KIF4A, and *RRM2,* that may be involved in tumorigenesis and in the processes of invasion and progression of disease. Array findings were validated using qRT-PCR, immunohistochemistry, and extensive *in silico* analyses of independently performed microarray datasets. To our knowledge, this study provides the first comprehensive genomic analysis of breast cancer in Middle Eastern women in age-specific cohorts and potential markers for cancer progression in young women. Our data demonstrate that cancer appearing in young women contain distinct biological characteristics and deregulated signaling pathways. Moreover, our integrative genomic and cross-species analysis may provide robust biomarkers for the detection of disease progression in young women, and lead to more effective treatment strategies.

## Introduction

Breast cancer is the most common type of cancer among women worldwide with an estimated 1,300,000 new cases and 465,000 deaths annually [1]. Breast cancer is the major cause of morbidity and mortality among females in Saudi Arabia [2]. Clinical observations indicate that 45% of all female breast cancers in Saudi Arabia developed before the age of 45 years, compared to 9.6% in the United States of America [2], [3]. Breast cancer diagnosed in young women is more aggressive in nature with a poorer prognosis and disease free survival compared to older counterparts [4], [5], [6], [7]. Indeed, it has been shown that survival in younger women is significantly worse for all stages of breast cancer in comparison to older women [8], [9]. Although previous studies have described young age is an independent predictor of poor prognosis, the underlying biology driving the aggressive nature of breast cancer arising in young women remains to be elucidated [10], [11], [12], [13].

Typically, the most common histologic type of breast cancer initiates as a premalignant lesion known as atypical ductal hyperplasia (ADH), then progresses into the preinvasive stage called ductal carcinoma *in situ* (DCIS), and culminates in invasive ductal carcinoma (IDC) [14]. Though it is a multistep process during which genetic alterations accumulate, molecular and pathological evidence suggests that DCIS is a precursor to invasive disease [15], [16], [17], [18]. A genome-wide microarray-based gene expression analysis would be expected to provide an opportunity to discover genes specifically activated or inactivated during the course of breast cancer progression. Despite recent research, the mechanisms underlying tumorigenesis and progression of breast cancer in young women is still not clear [19], [20]. In particular, the identification of “progression markers” is crucial for determining which lesions are likely to become invasive.

A cross-species comparative genomics approach represents a powerful strategy to identify target genes that may play a role in tumor initiation and progression to malignancy and thus has great therapeutic potential [21], [22], [23], [24], [25]. Previous studies have used this approach successfully to understand the molecular pathogenesis of various cancers and disease progression [23], [24], [26], [27]. The rationale is that genomic aberrations and altered pathways involved in oncogenesis are conserved by evolution across different species [24], [26], [28], and a number of important driver mutations in various cancers have been identified using comparative genomic approaches [24], [28], [29]. For example, cross-species gene-expression analysis of mouse and human data uncovered gene expression signatures that demonstrate K-Ras oncogene activation in human lung cancers [24]. In another example, Scott Lowe and colleagues identified two oncogenes that are co-amplified and cooperate to promote tumorigenesis by comparing gene amplifications in mouse and human hepatocellular carcinomas [29].

There are areas of genomic instability reported in many cancers, including breast cancer, and some regions commonly exhibit either deletion or increased gene dosage, leading to changes in DNA copy number (CN) [30], [31], [32], [33]. Integrating gene expression with CN data is an effective strategy for interpreting DNA and RNA level anomalies in cancer to identify genes involved with tumor initiation and progression [33], [34], [35]. Hence, integrating cross-species comparative analysis of human and animal models of breast cancer progression with genomic DNA copy number alterations may lead to robust biomarkers for breast cancer disease progression [32], [33], [36], [37], [38].

In this study, we analyzed whole-genome mRNA expression profiling from breast tumors and adjacent normal tissues from Middle Eastern women (n = 113 samples) in age-specific cohorts to characterize the underlying biology of aggressive breast cancers appearing in young women. Moreover, we performed an integrative and cross-species comparative genomics approach to identify evolutionarily conserved marker genes for disease progression in young women and validated its prognostic potential.

## Materials and Methods

### Patients and Samples

In this study, we focused on breast cancer patients diagnosed with infiltrating ductal carcinoma (IDC) and ductal carcinoma *in situ* (DCIS). Breast cancer samples were collected from primary tumors of 76 patients who sought treatment and underwent surgery (breast conservation surgery or total mastectomy) at the King Faisal Specialist Hospital and Research Center. Signed informed consent was obtained from all patients. On excision of tissues by a surgeon, an anatomic pathologist obtained a sample of the tumor tissue and adjacent normal breast tissue from the same breast having the tumor. 113 samples were collected from patients and fully consented according to institutional review board approved protocols (KFSHRC IRB Protocol). The study was approved by the research ethics board at our institution (RAC# 2031091). Fresh surgical samples including tumors and adjacent disease free tissues were placed in RNAlater™ (Ambion, Inc) and stored at −20°C after micro dissection had been performed for pathological confirmation. All normal breast tissues were confirmed by the pathologist to have normal morphology before the results were analyzed. Whenever possible depending on the quantity of the surgical samples, a piece of every sample was also snap frozen in liquid nitrogen and then stored at −80°C for subsequent isolation of DNA and proteins. The majority of samples received no prior chemotherapy; only two had chemotherapy and were excluded from further analysis.

Histological assessment of tumors and axillary lymph nodes were done by using formalin-fixed, paraffin-embedded breast cancer samples for HER2, estrogen receptor (ER), and progesterone receptor (PR) status. ER status was determined by immunohistochemistry and measured as a percentage and intensity of positive nuclear staining. The estrogen and progesterone receptors were stained with relevant specific antibodies (Novocastra, Newcastle upon Tyne, UK). For HER2 immunohistochemistry, HercepTest™ (Dako Denmark A/S, Glostrup, Denmark) was used with scores of 0 and 1+ considered negative and 2+ equivocal and 3+ considered positive.

Cancers were categorized as luminal A (ER-positive and/or PR-positive and HER2- and either histologic grade 1 or 2); luminal B (ER-positive and/or PR-positive and HER2+ or ER-positive and/or PR-positive, HER2- and grade 3); HER2 (ER-negative and PR-negative and HER2+); and triple negative (ER-, PR-, and HER2-) as defined previously [39]. Description of the clinicopathological characteristics of patients and breast cancer subtypes for luminal A, luminal B, HER2, and triple negative based on the histological evaluations are shown in Table 1.

**Table 1:** Age-specific patients’ characteristics.

Characteristic	All Patients No (%)	Very young (≤35) No (%)	Young (35–45) No (%)	Pre (45–55) No (%)	Old (≥55) No (%)
**Type**					
IDC	64(90.1)	5(83.3)	24(85.7)	12 (92.3)	23(95.8)
DCIS	7(9.9)	1(16.7)	4(14.3)	1 (7.7)	1(4.2)
**Normal**	33 (100)	3(9.1)	15(45.5)	7(21.2)	8(24.8)
**ER**					
positive	49 (69.0)	6(100.0)	15(53.6)	9(69.23)	19(79.2)
Negative	18 (25.4)	0(0)	10(35.7)	4(30.8)	4(16.7)
Missing	4 (5.6)		3(10.7)		1(4.2)
**PR**					
positive	38(53.5)	5(83.3)	13(46.4)	7(53.9)	13(54.2)
Negative	29(40.9)	1(16.7)	12(42.9)	6(46.1)	10(41.7)
Missing	4(5.6)		3(10.7)		1(4.2)
**Grade**					
1	3(4.2)	0(0)	0(0)	2(15.4)	1(4.2)
2	38(53.5)	5(83.3)	12(42.9)	7(53.9)	14(58.3)
3	24(33.8)	1(16.7)	12(42.9)	3(23.1)	8(33.3)
missing	6(8.5)	0(0)	4(14.3)	1(7.7)	1(4.2)
**HER2**					
Positive	33(46.5)	4(66.7)	13(46.4)	6(46.2)	10(41.7)
Negative	34(47.9)	2(33.3)	12(42.9)	7(53.9)	13(54.2)
Missing	4(5.6)		3(10.7)		1(4.2)
**Lymph Node**					
positive	38(53.5)	2(33.3)	13(46.4)	9(69.2)	14(58.3)
Negative	28(39.4)	3(50.0)	13(46.4)	3(23.1)	9(37.5)
Missing	5(7.0)	1(16.7)	2(7.1)	1(7.7)	1(4.2)
**LIVI**					
seen	35(49.3)	4(66.7)	10(35.7)	8(61.5)	13(54.2)
absent	31(43.6)	2(33.3)	15(53.6)	4(30.8)	10(41.7)
missing	5(7.0)	0(0)	3(10.7)	1(7.7)	1(4.2)
**Subtypes**					
Luminal A	24(33.8)	2(33.3)	7(25.0)	6(46.2)	9(37.5)
Luminal B	25(35.2)	4(66.7)	7(25.0)	3(23.1)	11(45.8)
HER2	14(19.7)	0(0)	8(28.6)	4(30.8)	2(8.3)
Triple negative	3(4.2)	0(0)	2(7.1)	0(0)	1(4.2)
Missing	5(7.0)	0(0)	4(14.3)	0(0)	1(4.2)

### Array Hybridization

Total RNA was extracted from tumor and adjacent normal tissue from patients with standard protocols. Sample handling, cDNA synthesis, cRNA labeling and synthesis, hybridization, washing, array (GeneChip® Human Genome U133Plus 2.0 Array, Affymetrix Inc., Santa Clara, CA, USA) scanning, and all related quality controls were performed according to the manufacturer’s instructions. The Affymetrix GeneChip/GCOS software (Affymetrix Inc.) was used to calculate the raw expression value of each gene from the scanned image. The total RNA quality was assessed by the values of the 3′–5′ ratios for actin and glyceraldehyde- 3-phosphate dehydrogenase (GAPDH). DChip [40], [41] outlier detection algorithm was used to identify outlier arrays. 104 samples/chips passed the above mentioned quality controls and were used for further analyses. The CEL files were utilized for further analysis using dChip [40], [41], MEV [42], [43], and PARTEK Genomics Suite (Partek® software, Partek Inc., St. Louis, MO, USA).

### Microarray Analysis

Global expression profiling of samples from tumor, IDC (n = 64) and DCIS (n = 7), and adjacent disease free tissues (n = 33) were probed using Affymetrix’s GeneChip® Human Genome U133 Plus 2.0 Arrays representing over 47,000 transcripts and variants using more than 54,000 probe sets. The open source R/Bioconductor packages, (Fred Hutchinson Cancer Research Center, Seattle, WA, USA) [44] were employed to normalize the data by the GC Robust Multi-array Average (GC-RMA) algorithm [45], [46]. The GC-RMA takes into account the GC content of the probe sequences when comparing the expression intensities of the different probe sets. To determine significant differences in gene expression levels among different age groups (young women (≤45 years), 45 to 55 years (pre) and ≥55 years (elderly) cohorts), we performed a multi-factor ANOVA including ER, PR, HER2, and grade status as additional factors in a linear additive model, as described previously [47]. We used tumor samples data with complete pathological reports in this model (n = 67). Additionally, we used all tumor and normal samples (n = 104), and performed two-way ANOVA by taking age (young, pre, and elderly), type (tumor or normal), as well as their interaction into the model [47]. In this model, we compared transcriptomes of the tumor tissue and normal tissue for each age group separately. Significantly modulated genes were defined as those with an absolute fold change >2.0 and adjusted p-value <0.05. Multiple hypothesis testing was controlled by applying the Benjamini-Hochberg false discovery rate (FDR) correction. Unsupervised two-dimensional hierarchical clustering using Euclidean distance as well as Pearson’s correlation with average linkage clustering was performed. Biological themes associated with the differentially expressed genes was identified by using DAVID Bioinformatics Resources [48], Expression Analysis Systematic Explorer (EASE) [49], and Ingenuity Pathways Analysis (IPA) 6.3 (Ingenuity Systems, Mountain View, CA). Using these bioinformatics tools, we were able to gain greater biological insights into activated or repressed functional processes and altered pathways in the disease pathogenesis compared to the listing of differentially expressed genes. Categorical variables and differences in rates between groups were analyzed using the χ^2^ test. The Fisher exact test was used when expected cell counts were less than 5 using the Monte Carlo method as implemented in SAS. A P-value of <0.05 was considered significant. Statistical analyses were performed by using SAS 9.2 (SAS Institute, Cary, NC), MATLAB (The MathWorks), and PARTEK Genomics Suite softwares. All microarray data reported here are MIAME compliant and have been submitted to the NCBI Gene Expression Omnibus (GEO) database (GSE29044), according to MIAME standards [50].

### Independent Datasets

For cross-species analysis, the murine markers of disease progression are taken from Kretschmer et al (Table S4 in [37], GSE21444). Online analysis tools and databases developed by Gyorff et al [51] containing gene expression data and survival information from over 1800 breast cancer patients were obtained and downloaded from Gene Expression Omnibus (GEO; http://www.ncbi.nlm.nih.gov/geo/). These were used to assess the prognostic potential of our gene signature (details of the datasets included in the database are given in the original publication [51]). In addition, The Cancer Genome Atlas (TCGA) data from breast invasive carcinoma (n = 536) and matched normal (n = 63) (https://tcga-data.nci.nih.gov/tcga/), and datasets from GSE7390 [52] and GSE12093 [53] through the canEvolve web portal (www.canevolve.org/) were used for independent validation analyses. The Miller et al. [54] dataset (GSE3494) was also reanalyzed for validation of our gene signature. The GeneSigDB database [55] was used to find the overlap/overrepresentation of our gene signatures with previously published gene signatures for various cancers, including breast cancer. Finally, multiple large genomic data sets with DNA copy number alterations associated with breast cancer were retrieved from the Gene Expression Omnibus database through canEvolve (GSE7545, GSE16619, and GSE9154 data sets) and cBio Cancer Genomics Portals [56] (TCGA, Nature 2012 data [30]) for integrative genomic analysis.

### Functional Pathway and Network Analysis

Functional pathway, gene ontology and network analyses were executed using Ingenuity Pathways Analysis (IPA) 6.3 (Ingenuity Systems, Mountain View, CA), a web-delivered application that enables the discovery, visualization, and exploration of molecular interaction networks in gene expression data. The differentially expressed gene lists were mapped to their corresponding gene objects in the Ingenuity pathway knowledge base. These so-called focus genes were then used as a starting point for generating biological networks. A score was assigned to each network in the dataset to estimate the relevance of the network to the uploaded gene list. This score reflects the negative logarithm of the P that indicates the likelihood of the focus genes in a network being found together due to random chance. Using a 99% confidence level, scores of *≥*2 were considered significant. A right-tailed Fisher’s exact test was used to calculate a p value determining the probability that the biological function (or pathway) assigned to that data set is explained by chance alone.

### Realtime RT-PCR Experiments

Confirmatory realtime RT-PCR experiments were performed using the ABI 7500 Sequence Detection System (Applied Biosystems). 50 ng total RNA procured from the same microarray study samples were transcribed into cDNA using a Sensicript Kit (QIAGEN Inc., Valencia, CA, USA) under the following conditions: 25°C for 10 min, 42°C for 2 hrs, and 70°C for 15 min in a total volume of 20 µl. Five differentially expressed genes (*ESR1, IL1RN, SEPP1, TIAM1,* and *SCD*) were selected and primers designed using Primer3 software. After primer optimization, realtime PCR experiments were performed with 6 µl cDNA using Quantitech SyBr Green Kit (QIAGEN), employing GAPDH as the endogenous control gene. All reactions were conducted in triplicates and the data was analyzed using the delta delta C_T_ method [57], [58].

### Immunohistochemistry

Validation of protein expression was done using immunohistochemistry. Immunohistochemical staining was performed using standard techniques. Monoclonal anti-TGF-α antibody (Calbiochem, clone 213-4.4, dilution 1∶50), monoclonal anti PI3 kinase P85 alpha antibody (Abcam, Cambridge, UK, clone ep380y, dilution 1∶20) and polyclonal anti IL1 Receptor I antibody (Abcam, Cambridge, UK, Protein G purified, dilution 1∶20) were run manually. Slides were deparaffinized by routine techniques. Antigen retrieval was done in Tris/EDTA buffer, pH 9 heated at 95°C in a microwave for 25 minutes. After blocking endogenous peroxidase activity with a 3% aqueous H_2_O_2_ solution for 5 minutes, the sections were incubated with primary antibodies overnight at 4°C. Labeling was detected with Envision Plus Detection Kit (Dako, cat. No. K4001). Reaction was detected either by DAB (3, 3-diaminobenzidine, sigma, cat. No. D5905-100TAB) or by AEC (3- amino-9-ethylcarbozale, sigma, cat. No. A-5754). The sections were counterstained with Harris hematoxylin (Acros Organics). Staining was visualized using the DAKO Envision kit according to the instructions of the manufacturer (DAKO, Carpinteria, CA).

## Results

### Global Expression Profiling in Different Age Cohorts

Genome-wide gene expression profiling provides a comprehensive view of the transcriptional changes that occur during the carcinogenic process and enables the understanding of biology beyond what may be apparent from studies assessing only clinicopathologic features. Here, we first analyzed the whole-genome mRNA expression profile from tumors (n = 71) and adjacent disease free tissues (n = 33) and compared tumor with the normal tissue in each age cohort, young women (≤45 years), 45 to 55 years (pre) and ≥55 years (elderly), separately. We identified 2632, 2029 and 2842 significantly dysregulated genes (up- or down-regulated) present in tumors from young, pre and elderly cohorts (adjusted p value <5% and FC >2), respectively (Figure S1A). To obtain deeper insight into tumor pathogenesis in each age cohort, we performed gene ontology (GO) enrichment and interaction network analyses by using Expression Analysis Systematic Explorer (EASE) [49] and the Ingenuity knowledge base. The network analysis indicated activation of MYC, NF-κB and TGF-β signaling pathways in young, pre and elderly cohorts, respectively (Figure S1B).

### Genomic Signature Specific to Tumors Arising in Young Women

**Figure 1:**
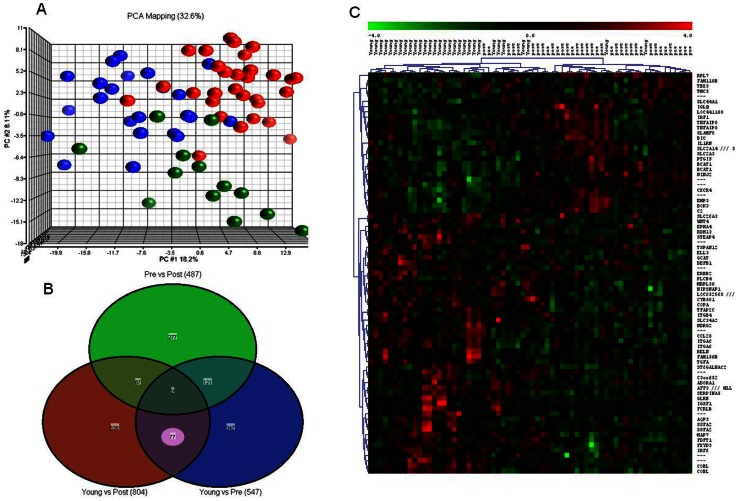
Identification of genes specific to young women with breast cancer. (**A**)The unsupervised principal component analysis (PCA) separated samples according to their age group hence supporting the conclusion that there is a distinct gene expression changes associated with the tumor in different age groups. The red spheres refer to young patients (≤45; Young), green for 45–55 years (Pre), and blue for ≥55 years (Post). (**B**) Venn diagram characterizing differential gene expression between and specific to different age groups. The red circle (left) shows the 804 probes that are differentially expressed between Young and Post; 77 probes (corresponding to 63 genes) were found to be specific to tumor in young women only (circled in *light pink)*. **(C**) Unsupervised two-dimensional hierarchical clustering of all tumor samples based on their gene expression similarity using young-age-specific 77 probes was performed using Pearson’s correlation with average linkage clustering. The hierarchical clustering revealed clear pattern of genes deregulation defining two main transcriptome clusters, one was mainly composed primarily younger cases, and one was composed of primarily elderly women. Samples are denoted in columns and genes are denoted in rows (gene symbols listed on the right). The expression level of each gene across the samples is scaled to [−4, 4] interval. These mapped expression levels are depicted using a color scale as shown at the bottom of the figure, as such highly expressed genes are indicated in red, intermediate in black, and weakly expressed in green.

We next compared the transcriptomes of tumors across three age cohorts using a multi-factor ANOVA, controlling for ER, PR, HER2, and grade of the tumors (n = 67). The ANOVA identified 567 genes that were significantly modulated among three age groups (unadjusted p<0.01). The unsupervised principal component analysis (PCA) using 567 genes separated samples according to their age group, hence supporting the conclusion that there are distinct gene expression changes associated with tumors that are dependent on the age of the patient (Figure 1A). We then analyzed overrepresentation of any clinicopathologic or tumor subtype among the age groups, and found no statistically significant associations.

**Figure 2:**
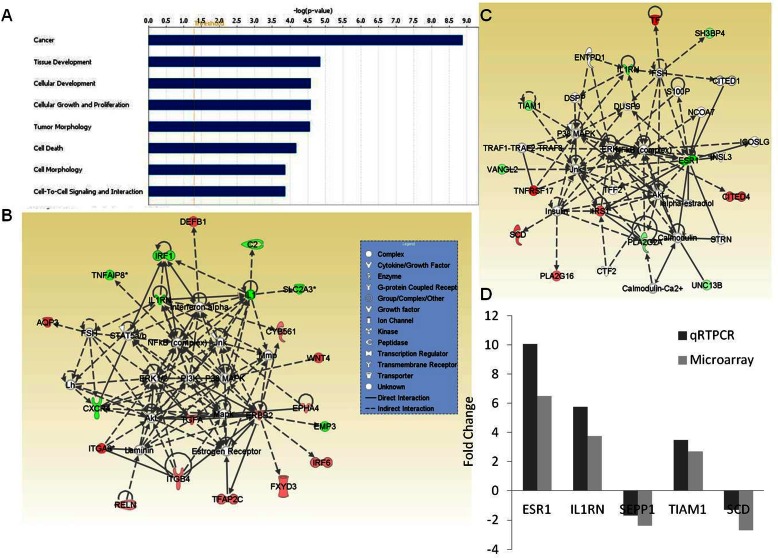
Functional and network analyses of genes specific to young women. (**A**) The gene ontology and functional analysis of young-age-tumor specific genes (up/down-regulated) were performed using the Ingenuity knowledge base. X-axis indicates the significance (-log P value) of the functional/pathway association that is dependent on the number of genes in a class as well as biologic relevance. The threshold line represents a P value of 0.05. (**B–C**) Gene interaction network analyses of genes specific to young women and very young women, respectively. Top scoring gene interaction networks with high relevancy scores (with highest relevance score) are shown. Green/red indicates decreased/increased mRNA expression in younger patients compared to older counterparts. The color intensity is correlated with fold change. Straight lines are for direct gene to gene interactions, dashed lines are for indirect ones (**D**) QRTPCR validation. Grey bars represent microarray hybridizations, and, and dark bars represent values from qRT-PCR. Ratio of expression for each gene in older group (>45) to very young group (≤35) is shown as fold change. A significant correlation existed between the microarray and realtime RT-PCR results.

The gene signature specific to tumors in young women (≤45 years) were obtained by overlapping gene lists. When comparing two groups of samples to identify genes differentially expressed in a given group, we used p-value and the fold change (FC) between two groups as the cut-off criteria. As shown in Figure 1B, each circle in the Venn diagram represents the differential expression between two “age groups”. This Venn diagram approach revealed that 79 probes were common to both ≤45 vs 45–55 and ≤45 vs >55 comparisons, and 77 probes (corresponding to 63 genes) were specific to tumors in the young group of patients (Y) (shown in pink, in Figure 1B, listed in Table 2) that have significantly higher or lower expression in young women compared to their older counterparts. The unsupervised two-dimensional hierarchical clustering using 63 genes revealed clear patterns of gene deregulation defining two main transcriptome clusters, one was mainly composed of primarily younger women, and the other one was composed primarily of older patients (Figure 1C). The Microarray Literature-based Annotation (MILANO) database [59] search indicated 98% of those 63 genes had a published association with cancer. Moreover, we tested 63 young age-specific gene signatures against the published gene signatures in GeneSigDB database [55], and found overrepresentation of our gene set in over 500 gene signatures for various cancers, including breast cancer (adjusted p-value <0.05). The GO and functional analyses revealed significant enrichment of categories, including carcinogenesis, tissue development, cellular development, cellular growth and proliferation, tumor morphology, and cell death (Figure 2A). The network analysis indicated alterations in a number of cancer related pathways, including p38 MAPK, PI3K/AKT, ERK/MAPK and NF-κB signaling pathways, and a potential role of TGFA, ErbB2, and IL-1/IL-1R in young women with breast cancer (Figure 2B).

**Table 2:** Differentially expressed genes between young women and two older cohorts.

Gene Symbol	Gene Title	FC^a^	FC^b^
***Genes with significantly higher expression in the young cohort***			
*FCRLB*	Fc receptor-like B	3.71	3.62
*COBL*	cordon-bleu homolog (mouse)	3.37	2.91
*GLRB*	glycine receptor, beta	2.74	2.18
*ITGA6*	integrin, alpha 6	2.64	2.40
*AQP3*	aquaporin 3 (Gill blood group)	2.45	3.25
*DEFB1*	defensin, beta 1	2.33	4.06
*SERPINA5*	serpin peptidase inhibitor, clade A (alpha-1 antiproteinase, antitrypsin), membe	2.30	4.24
*SLC26A3*	solute carrier family 26, member 3	2.27	2.82
*WNT4*	wingless-type MMTV integration site family, member 4	2.21	2.77
*IGSF1*	immunoglobulin superfamily, member 1	2.14	2.15
*TGFA*	transforming growth factor, alpha	2.08	2.75
*PLCB4*	phospholipase C, beta 4	2.06	2.17
*ERBB2*	v-erb-b2 erythroblastic leukemia viral oncogene homolog 2, neuro/glioblastoma de	2.00	2.99
*NDRG2*	NDRG family member 2	1.96	2.07
*MRPL30*	mitochondrial ribosomal protein L30	1.93	1.54
*AFF3/MLL*	AF4/FMR2 family, member 3///myeloid/lymphoid or mixed-lineage leukemia (tritho	1.93	2.19
*ST6GALNAC2*	ST6 (alpha-N-acetyl-neuraminyl-2,3-beta-galactosyl-1,3)-N-acetylgalactosaminide	1.90	2.32
*CCL28*	chemokine (C-C motif) ligand 28	1.85	3.23
*FAM150B*	family with sequence similarity 150, member B	1.84	1.88
*FXYD3*	FXYD domain containing ion transport regulator 3	1.80	1.99
*TFAP2C*	transcription factor AP-2 gamma (activating enhancer binding protein 2 gamma)	1.79	1.81
*IRF6*	interferon regulatory factor 6	1.77	1.88
*COPA*	coatomer protein complex, subunit alpha	1.77	1.87
*ITGB4*	integrin, beta 4	1.71	1.83
*STEAP4*	STEAP family member 4	1.70	1.68
*MAP7*	microtubule-associated protein 7	1.69	1.53
*SLC34A2*	solute carrier family 34 (sodium phosphate), member 2	1.68	1.83
*RELN*	reelin	1.68	1.80
*C3orf52*	chromosome 3 open reading frame 52	1.68	1.97
*RDH13*	Retinol dehydrogenase 13 (all-trans/9-cis)	1.67	1.56
*ADORA1*	adenosine A1 receptor	1.65	2.64
*TSPAN12*	tetraspanin 12	1.65	2.07
*NIPSNAP1*	nipsnap homolog 1 (C. elegans)	1.62	1.57
*ELL3*	elongation factor RNA polymerase II-like 3	1.61	2.12
*SSFA2*	sperm specific antigen 2	1.60	1.78
*EPHA4*	EPH receptor A4	1.58	2.22
*GCAT*	glycine C-acetyltransferase (2-amino-3-ketobutyrate coenzyme A ligase)	1.58	1.95
*FDFT1*	farnesyl-diphosphate farnesyltransferase 1	1.55	1.62
*CYB561*	cytochrome b-561	1.55	1.59
***Genes with significantly lower expression in the young cohort***			
*DOK3*	docking protein 3	−1.52	−1.54
*TBX3*	T-box 3	−1.55	−2.21
*EMP3*	epithelial membrane protein 3	−1.58	−1.64
*BIC*	BIC transcript	−1.61	−1.75
*SLC44A1*	solute carrier family 44, member 1	−1.61	−1.72
*C2*	complement component 2	−1.62	−1.60
*LOC441108*	hypothetical gene supported by AK128882	−1.63	−1.66
*NINJ2*	ninjurin 2	−1.64	−2.04
*IRF1*	interferon regulatory factor 1	−1.64	−1.70
*SLAMF8*	SLAM family member 8	−1.66	−1.87
*RPL7*	ribosomal protein L7	−1.68	−1.51
*CXCR4*	chemokine (C-X-C motif) receptor 4	−1.70	−1.76
*PTGIS*	prostaglandin I2 (prostacyclin) synthase	−1.72	−2.08
*TNFAIP8*	tumor necrosis factor, alpha-induced protein 8	−1.73	−2.01
*SLC2A14/SLC2A3*	solute carrier family 2 (facilitated glucose transporter), member 14///solute	−1.74	−1.70
*SLC2A3*	solute carrier family 2 (facilitated glucose transporter), member 3	−1.77	−1.73
IGL@	immunoglobulin lambda locus	−1.86	−1.79
*BCAT1*	branched chain aminotransferase 1, cytosolic	−1.97	−2.30
*FAM110B*	family with sequence similarity 110, member B	−2.08	−2.82
*TMC5*	transmembrane channel-like 5	−2.15	−2.70
*IL1RN*	interleukin 1 receptor antagonist	−2.34	−1.82

^a^FC was calculated between the mean values of expression observed in young women (≤45 years) and ≥55 years.

^b^FC was calculated between the mean values of expression observed in young and 45–55 years.

### Genomic Signature Specific to Breast Cancers in Very Young Woman

In Saudi Arabia, almost 50% of all the breast cancer patients were reported to be less than 45 years old. Accordingly, we performed additional analyses within the young women’s subset comparing transcriptomes of women younger than 35 years (very young) to two other age cohorts: 35 to 45 years and >45 years. We identified genes that were specific to tumors in very young women using the same methodology that was described previously. The heat map clearly shows significantly higher or lower expression of these genes in very young women compared to the two older age cohorts (Figure S2). The enriched biological processes associated with significantly dysregulated genes that are unique to very young patients include, among others, mitotic cell cycle (p-value = 0.02), morphogenesis (p-value = 0.01), cell proliferation (p-value = 0.03), and death (p-value = 0.049). Similar to young women, network analysis indicated alterations in p38 MAPK, PI3K/AKT and NF-κB signaling pathways, and potentially important roles of IL1RN, ESR1, and ErbB2 in very young women (Figure 2C and Figure S2).

### Cross-Species Comparative Genomics Analysis Coupled with Genomic Alteration Data to Identify Genes that may Play a Role in Cancer Development and Progression in Young Women

**Figure 3:**
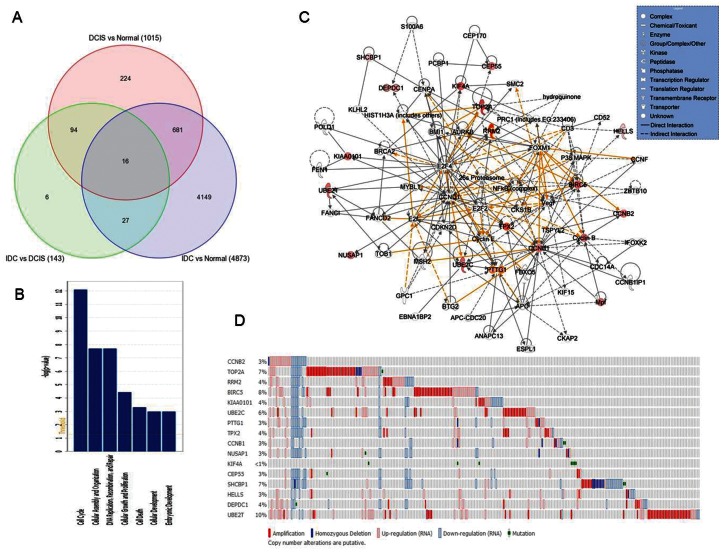
Progression from ductal carcinoma in situ (DCIS) to invasive ductal carcinoma (IDC) in young women. (**A**) The Venn diagram illustrates that there are 1015 genes differentially expressed (up- or down-regulated) in DCIS compared to normal, whereas 4873 genes differentially expressed in IDC compared to normal controls. 143 genes differentially regulated between IDC and DCIS (green circle). (**B**) The functional analysis of 16 potential progression genes identified through cross-species comparative genomics analysis. Y-axis indicates the significance (-log P value) of the functional association that is dependent on the number of genes in a class as well as biologic relevance. The threshold line represents a P value of 0.05. (**C**) Gene interaction networks and pathways analyses of 16-gene progression signature. Green/red indicates decreased/increased mRNA expression in IDC compared to normal controls. The color intensity is correlated with fold change. Straight lines are for direct gene to gene interactions, dashed lines are for indirect ones. (**D**) Invasive breast tumor cases (from TCGA, Nature 2012 [30]) displayed altered amplification/homozygous deletion/up-or down-regulation (RNA) or mutation in our 16-progression gene signature. Cases are denoted in columns, and genes in rows (gene symbols are listed on the left).

Ductal carcinoma in situ (DCIS) is heterogeneous group of pre-invasive tumors which may progress rapidly or slowly to invasive cancer. Therefore, an ability to identify which DCIS lesions are likely to progress to the potentially life threatening stage of invasive ductal carcinoma (IDC) would greatly help in the treatment plan and prognosis of the disease. To identify the putative genes involved in disease progression in young women, we performed genome-wide gene expression profiles characteristic of the sequential disease stages (DCIS and IDC) of breast cancer and compared them to age-matched normal controls in young women (≤45 years). We defined potential progression genes as genes that are significantly altered in both DCIS and IDC as these likely represent the earliest molecular steps in acquiring the capacity for invasion [15], [19], [37]. We identified 1015 and 4873 genes differentially expressed (up and down-regulated) in DCIS and IDC compared to normal, respectively, and 697 probes (corresponding to 484 unique genes) that had significantly altered expression in both DCIS and IDC (Figure 3A).

We next performed cross-species comparative genomics analysis to identify potential gene markers for DCIS progression to IDC that are conserved in mouse and human. This approach has been shown to lead to robust markers that may play a role in cancer development and progression. Indeed, driver mutations that are important in cancer have been identified using this strategy [23], [24], [25], [27], [29], [36]. We used gene expression data from Kretschmer et al. [37] for murine markers of disease progression. The comparison of our progression gene signature with the murine markers (human orthologous) revealed 16 genes that were conserved between mouse and human (p<0.001) (Table 3). GO analyses using both EASE and IPA tools revealed that these genes are mainly involved in biological processes such as cell cycle, mitosis, embryonic development, DNA replication, growth and apoptosis (Figure 3B). The top five significantly altered canonical pathways include Cell cycle: G2/M DNA Checkpoint Regulation (p value = 1.1×10^−5^), Mitotic Roles of Polo-Like Kinase (p value = 3.3×10^−5^), ATM Signaling (p value = 1.5×10^−3^), Cyclins and cell cycle regulation (p value = 2.9×10^−3^), and Sonic hedgehog signaling (p value = 0.03). The network analysis illustrated activated pathways as well as interactions of genes that may potentially play a role in disease progression (Figure 3C). A literature-based search of 16 genes using the MILANO database [59] demonstrated the association of these genes with cancer progression, tumor development and invasion in various cancers, including breast cancer [22], [37], [60], [61], [62], [63], [64].

**Table 3:** List of 16 cross-species conserved DCIS to IDC potential progression gene signature.

Gene^1^	Gene Name	DCIS^a^	IDC^b^	Biological process term	HR (95%CI)^c^	p-value^d^	GSE7390†	GSE12093^‡^
CCNB2*	cyclin B2	4.83	4.76	embryonic development; cell cycle; mitosis; growth	2.23(1.94–2.56)	<1e-16	7.6e-4	0.016
TOP2A*,**	topoisomerase (DNA) II alpha 170kDa	8.38	7.9	DNA metabolic process; DNA replication	2.21(1.92–2.54)	<1e-16	5.8e-3	2.9e-3
RRM2*	ribonucleotide reductase M2 polypeptide	23.96	10.4	DNA replication	2.01(1.75–2.31)	<1e-16	0.01	4.1e-4
BIRC5*	baculoviral IAP repeat-containing 5	7.28	6.10	G2/M transition of mitotic cell cycle; mitosis; cell division	2.07 (1.8–2.38)	<1e-16	0.03	4.7e-3
KIAA0101*	KIAA0101	4.76	5.12	–	1.78 (1.55–2.05)	1.1E-16	NS	7.5e-3
UBE2C*	ubiquitin-conjugating enzyme E2C	4.98	5.83	ubiquitin-dependent protein catabolic process; cell cycle; mitosis; protein ubiquitination	1.81 (1.58–2.08)	<1e-16	0.02	NS
PTTG1*	pituitary tumor-transforming 1	4.23	3.76	DNA metabolic process; DNA repair; cell cycle; mitosis; cell division	1.71 (1.49–1.96)	8.1E-15	NS	2.9e-3
TPX2*	TPX2, microtubule-associated, homolog (Xenopus laevis)	6.95	7.28	apoptosis; cell cycle; mitosis; cell proliferation; cell division	1.83 (1.59–2.1)	<1e-16	0.01	0.014
CCNB1*,**	cyclin B1	5.63	4.85	G2/M transition of mitotic cell cycle; mitotic cell cycle; inutero embryonic development; cell division cycle	2.36 (2.05–2.72)	<1e-16	NS	0.02
NUSAP1*,**	nucleolar and spindle associated protein 1	7.40	4.85	cell cycle; mitosis; cell division	1.82 (1.59–2.09)	<1e-16	0.02	0.04
KIF4A**	kinesin family member 4A	5.41	6.4	organelle organization; microtubule-based movement	1.91 (1.66–2.19)	<1e-16	0.01	NS
CEP55*,**	centrosomal protein 55kDa	7.27	4.7	cell cycle; mitosis; cell division	2.2 (1.92–2.53)	<1e-16	NS	0.04
SHCBP1*,**	SHC SH2-domain binding protein 1	3.72	2.17	–	1.87 (1.63–2.14)	<1e-16	0.048	NS
HELLS*	helicase, lymphoid-specific	4.11	2.05	cell cycle; mitosis; multicellular organismal development; lymphocyte proliferation; cell division	1.74 (1.51–1.99)	1.7e-15	NS	NS
DEPDC1*,**	DEP domain containing 1	5.61	3.50	signal transduction	1.26 (1.1–1.44)	7.5e-4	NS	NS
UBE2T*	ubiquitin-conjugating enzyme E2T (putative)	5.83	5.56	DNA repair, protein monoubiquitination; response to DNA damage stimulus	na	na	na	na

^1^Genes with asterisk are also located in the chromosomal CNA region and ** Mutation found in patients in [30].

^a^DCIS indicates fold change between the mean values of expression observed in DCIS (ductal carcinoma *in situ*) and age-matched normal controls.

^b^IDC indicates fold change between the mean values of expression observed in IDC (invasive ductal carcinoma) and age-matched normal controls.

^c^Hazard ration (HR) with 95% confidence intervals and ^d^logrank P-value for Recurrence free survival (RFS) using data from Gyorfffy et al [51].

^†^ and ^‡^ logrank P-value for distant metastasis free survival using data from GSE7390 [52] and GSE12093 [53], respectively. NS. Not significant; na:Not available.

The presence of altered DNA CN may contribute to cancer formation and progression and could include transcriptional control mechanisms that locally impact gene expression levels [31], [33], [65], [66]. Integrating the gene expression data with CN alterations may identify novel early breast cancer markers of malignant transformation and progression [33], [34], [35]. Hence, we integrated our cross-species conserved progression gene signature with four independent studies of genome copy number alterationsin human breast tumors (as detailed in “Materials and Methods” section) and found that our gene signature has concomitant DNA alterations [30], [31], [32] (Table 3, Figure 3D).

### Comparison of DCIS and IDC Transcriptome in Young Women

Comparison of expression profile characteristics between IDC and DCIS in young women revealed dysregulation of 143 genes, 96% of which had significantly higher expression in DCIS compared to those in IDC (Figure 3A). These genes were enriched within functional categories including immune response, tissue morphology, cellular growth and proliferation, cell death and cellular movement. The network analysis highlighted alterations in PI3K/Akt, NFkB, Jnk, and ERK pathways (Figure S3).

The Venn diagram approach resulted in 27 genes and 94 probes (corresponding to 72 genes) that were unique to IDC and DCIS, respectively (Table S1, Figure S3). Interestingly, 85% of genes specific to IDC were down-regulated compared to normal controls. The IDC gene signature, including *DUSP6, PTGDS, IFNGR1, PIK3R1, FCER1A, P2RY14, PVRL2, SELP*, and TFPI were involved in cell death, immune response, cellular movement and tissue development. The interaction network and pathway analyses revealed alterations in G-Protein Coupled Receptor Signaling, PI3K Signaling, and ERK/MAPK Signaling (Figure S3). In contrast to IDC, 97% of DCIS specific genes were up-regulated in DCIS vs normal, including genes such as *CD22, IGHM, MS4A1, BCR, RBL2,* and *MAP3K5* (Table S1 and Figure S3).

### *In silico* Independent Validations

To validate our results, we used four independently performed microarray datasets as well as data available in the database developed by Gyorffy et al. [51]. The first validation dataset was generated by The Cancer Genome Atlas ((https://tcga-data.nci.nih.gov/tcga/). This dataset is composed of samples from invasive breast carcinoma patients (n = 536) and matched normal controls (n = 63). Our cross-species conserved 16-progression gene signature was significantly up-regulated in patients compared to normal controls (adjusted P-value <1.19×10^−32^) and was sufficient to cluster and differentiate samples as tumor versus normal controls (data not shown).

We then assessed the prognostic capability of our genes on independent microarray datasets involving large numbers of breast cancer patients with survival data. We confirmed the prognostic significance of all of our 16 genes for recurrence free survival (RFS;n = 2324), overall survival (OS;n = 464), and distant metastasis free survival (DMFS; n = 673) in datasets from Gyorffy et al. [51]. The high expressions of these genes were significantly associated with poor disease outcome (Table 3). Moreover, the prognostic significance of 16 genes were tested on additional two datasets of breast cancer patients from GSE7390 [52] and GSE12093 [53]. The GSE7390 dataset consisted of 198 lymph node-negative (N-) patients [52]. The purpose of this analysis was to identify patients at high risk of early distant metastases. The data from Zhang et al (GSE12093) [53] included 136 breast cancers that were treated with tamoxifen to classify high-risk patients that benefit from adjuvant tamoxifen therapy. We found that thirteen of our genes were significantly associated with a high risk patient group with distant metastases in at least two of the datasets tested (Table 3). Six genes (RRM2, BIRC5, TOP2A, NUSAP1, TPX2, and CCNB2*)* were of significant clinical relevance in all the datasets tested, especially for identifying a high risk patient group (Table 3, Figure S4).

As a further validation of our results, we re-analyzed an independently performed microarray dataset from Miller et al [54].This dataset was composed of 251 human breast cancer samples, of which 31 were derived from young women, which were used in this re-analysis. We evaluated the performance of the 16-progression gene signature on this dataset. Unsupervised clustering was performed and we found that our gene signature was sufficient to separate patients into two clusters which differed significantly by p53 mutation status (Figure S4). The cluster which had high expression of these genes comprised nearly of all the p53 mutant tumors. Intriguingly, TP53 mutations in breast cancer are associated with poor survival independent of other risk factors [67].

The Microarray Literature-based Annotation (MILANO) database [59] search revealed that all of the 16 genes were associated with tumor progression, development, and invasiveness in various cancers, including breast cancer [60], [61], [62], [63]. Moreover, comparing the 16-gene signature with gene signatures available in the GeneSigDB database [55] revealed statistically significant overlap (P-value <0.05, corrected for multiple testing) with over 400 published cancer gene signatures for various cancers, including 161 gene signatures for breast cancer. Furthermore, these genes were also mapped to human genomic CN alterations associated with invasive breast tumors in independent genomic studies, implicating the involvement of these genes in malignant transformation and progression [33], [34], [35].

### Validation of Microarray Data by qRT-PCR and Immunohistochemistry

To confirm the microarray results by an independent method, we selected five significantly dysregulated genes (ESR1, IL1RN, SEPP1, TIAM1, and SCD*)* in very young (≤35 years) and/or young (≤45 years) women compared to older cohorts and validated the expression levels using qRT-PCR. A significant correlation existed between the microarray and realtime RT-PCR results, (Figure 2D and Figure S2 (Pearson’s r >0.76). This correlation was stronger when comparing the older group (>45 years) to the very young women cohort (≤35 years) (r = 0.99; Figure 2D) versus comparing the young woman group (35–45 years) to the very young women cohort (r = 0.77; Figure S2).

**Figure 4:**
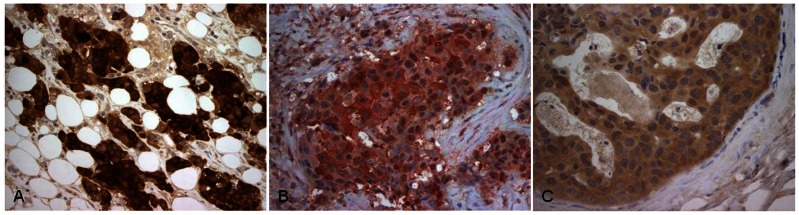
Protein expression of selected genes by immunohistochemical staining in breast cancer patients’ samples using antibodies directed against (B) TGFA, (C) IL1RN, and (D) PI3K. Representative images of positively stained tumors are shown (magnification, ×200).

Moreover, we performed immunohistochemical staining in breast cancer patient samples using antibodies directed against TGFA, IL1RN and PI3K. The TGFA positivity was significantly associated with young age (Fisher’s exact test, p value = 0.02). In fact, 90% of young patients (n = 10) tested positive, which is in concordance with the microarray result. IL1RN was found to have higher expression in older cohorts compared to young patients in our microarray analysis, which was also validated by qRT-PCR (Figure 2D). Indeed, five of the six samples that tested positive by immunohistochemical staining were from older patients. Testing for protein expression of PI3K revealed that it was not expressed in all of the IDC cases (n = 10), but positive for DCIS, which is also in concordance with the microarray result (Figure S3). Hence, the immunohistochemistry verified the protein expression of the selected candidates. Representative images of positively stained tumors are shown in Figure 4A–C, respectively).

## Discussion

Numerous studies have shown that younger women with breast cancer have a poorer prognosis and disease free survival compared to their older counterparts [4], [5], [6], [7], [13], [68]. Indeed, young age has been shown to be an independent predictor for poor prognosis even after controlling for different histopathological features [13], [69]. However, the biology driving this disease process and the molecular pathways that contribute to aggressive tumors in younger women are largely unknown. Clinical observations indicate that 45% of all female breast cancers in Saudi Arabia appear in women younger than 45 years of age [2]. Hence, in this study, we sought to understand the molecular underpinnings of breast cancer in an age-specific manner in order to elucidate genes and pathways giving rise to aggressive tumors in young women using a transcriptomic approach. Furthermore, we explored molecular alterations of breast cancer progression from DCIS to potentially lethal stages of IDC in young women and identified potential progression marker genes using cross-species comparative genomics analysis.

We performed two different approaches to identify gene signatures for different age cohorts of women with breast cancer. In the first approach, we compared whole-genome mRNA expression profile from tumors and disease free normal tissues in three age cohorts of young women (≤45 years), 45 to 55 years (pre) and ≥55 years (elderly). The network analyses of significantly dysregulated genes revealed the activation of MYC [68], [70], [71], NF-κB [72] and TGF-β signaling [73], [74] pathways in young, pre and elderly cohorts, respectively. In the second approach, we compared transcriptomes of tumors arising in young women to those from two older counterparts, and identified 63 genes that had distinct expression patterns in young women. By performing these approaches, we gained important insights into pathways and genes that were specifically altered in young women. The pathway analysis indicated alterations in PI3K/Akt [75], [76], MYC [68], [70], [71] and NF-κB [72] signaling pathways, and potential critical roles for *TGFA* [77], [78], *ErbB2* [7], [79], [80], [81], and *IL-1/IL-1R* [82], [83], [84] which may promote angiogenesis, tumor growth, and metastasis and hence cause the aggressive phenotype observed in young women. Previous reports have shown in experimental models that Interleukin 1 (*IL-1*) promotes angiogenesis, tumor growth, and metastasis [85], and its presence in some human cancers is associated with aggressive tumor biology [86]. The activation of *IL-1/IL-1R* though autocrine or paracrine mechanisms can lead to a cascade of secondary tumorigenic cytokines, which can subsequently contribute to angiogenesis, tumor-cell proliferation and tumor invasion [82]. For example, these inflammatory cytokines can regulate the proliferation of breast cells through estrogen production by the steroid catalyzing enzymes in breast tissues [87]. Interestingly, mutant alleles of *IL1RN* were associated with shortened disease-free and overall survival among Caucasian women with breast cancer [83]. Similarly, *IL-1* expression has been shown to be an adverse prognostic factor [84], [88]. NF-κB signaling has been shown to be activated in various tumors, including human breast cancers. Most recently, it has been shown in mouse models that epithelial NF-κB is an active contributor to tumor progression, inhibition of which could have a significant therapeutic impact even at later stages of mammary tumor progression [89]. Our data also indicated that the levels of expression of *TIAM1* and *VANGL2* in very young women are significantly lower than in their older counterparts. The expression of *TIAM1* has been shown to be associated with increased invasiveness and progression of breast carcinomas [90]. Recently, it has been reported that *VANGL2* promotes migration of cells by a metalloproteinase-dependent invasion of extra cellular matrix and therefore influences invasion and perhaps metastasis [91].

Previous studies have shown that important driver mutations in various cancers can be identified using comparative genomic approaches [21], [23], [24], [28], [29]. Such studies suggest that the conserved changes across species may be mechanistically essential for cancer development and progression, and hence they may be critical targets for therapeutic intervention [22], [28], [92]. Therefore, focusing on differentially expressed genes derived from these comparative approaches along with concomitant altered DNA copy number changes may identify novel early breast cancer markers of malignant transformation and progression [33], [34], [35]. One of the major contributions of this study is the identification of 16 potential disease progression marker genes, including *CCNB2, UBE2C, TPX2, KIF4A, BIRC5, NUSAP1*, and *RRM2,* using integrative and cross-species comparative genomics analysis. These genes are related to mitosis, cell cycle, embryonic development, DNA replication, cell division and proliferation. Our findings are consistent with previously performed independent studies of breast cancer progression [15], [20], [37]. However, the novelty of our results is that genes identified in this study were evolutionarily conserved across species, and along with genomic alterations, and we provide evidence for the potential role of previously reported genes as well as new genes in the progression of young women’s breast cancer progression.

Testing our genes on independent microarray datasets using samples from over 3000 breast cancer patients demonstrated that high expression of these genes are significantly associated with poor outcome. Intriguingly, our 16-gene signature separated patients in Miller *et. al*.’s study into two clusters that differed significantly in their TP53 mutation status. The cluster which had high expression of these genes comprised nearly of all the p53 mutant tumors. Previous studies have reported that TP53 mutations in breast cancer are associated with poor survival independent of other risk factors [67] and have a strong association with hormone receptor negative, HER2+ and basal-like subgroups [93], [94]. Furthermore, a Microarray Literature-based Annotation database [59] search indicated the involvement of our 16 genes in tumor development, progression, and invasiveness in various cancers, including breast cancer [22], [37], [60], [61], [62], [63], [64]. Taken together, these observations suggest that the 16-progression-gene signature has the potential to classify tumors which may have invasive capacity and may be crucial for determining which lesions are more likely to become invasive.

Differential expression analysis of DCIS and IDC in young women revealed significant down regulation of PI3K, DUSP6, CD22, RB, BCR, MS4A1 (also known as CD20), and MAP3K5 as well as alterations in PI3K/Akt, NFkB, Jnk, and ERK pathways. The PI3K/Akt pathway is involved in regulation of cell proliferation and implicated in carcinogenesis [95]. The network analysis also indicated a central role of the retinoblastoma tumor suppressor (RB), which may be potentially important in tumor progression. This gene has been found to be functionally inactivated in the majority of human cancers, and aberrant in nearly half of breast cancers [96]. Deficiency in RB function compromises cell cycle checkpoints, and contributes to aggressive tumor proliferation [96]. Comparison of IDC and DCIS transcriptomes resulted in 27 signature genes that are unique to IDC, and differentiated from DCIS in young women. The majority of these genes (85%) were repressed (or down-regulated) compared to normal controls, except for few genes, such as Poliovirus receptor-related 2 (PVRL2, CD112). PVRL2 has been found to have enhanced expression in various tumors, and it has been suggested to have a role in tumor invasion and migration [97], [98].

In summary, to our knowledge this study provides the first comprehensive transcriptomic analysis of breast tumors that characterizes the underlying biological mechanisms in an age-specific manner in a cohort of Middle Eastern women, and coupled with an integrative cross-species comparative genomics approach has identified genes that could be potential biomarkers for tumor progression in young women. Our global expression profiling resulted in 63 genes that are specific to young women’s breast tumors. The network analyses illustrated the interaction of potential critical genes and the altered pathways associated with breast cancer that specifically appear in young women. The implication from these findings is that these genes may be contributing to the aggressive tumor behavior often present in these patients. Our results confirm previous studies as well as provide additional insights into young age (≤45 years) and very young age (≤35 years) specific oncogenic alterations that may be promoting tumorigenesis. Our cross species data analyses coupled with genomic copy number alterations may provide robust biomarkers for the detection of disease progression in young women and may lead to improved diagnosis and therapeutic options.

## Supporting Information

Figure S1 **(A)** Comparison of each age cohort, young women (≤45 years), 45 to 55 years (pre) and ≥55 years (post), with the age-matched normal controls. We identified 2632, 2029 and 2842 significantly dysregulated genes (up or down) due to tumor in young, pre and old cohorts respectively (adjusted p value <5% and FC >2). (B) Gene interaction networks analysis of differentially expressed genes associated with tumor in each age cohort. Green/red indicates decreased/increased mRNA expression in patients compared to age-matched normal controls. The color intensity is correlated with fold change. Straight lines are for direct gene to gene interactions, dashed lines are for indirect ones (top scoring networks are shown).
(DOCX)

Figure S2 **(A)** Heatmap of very young-specific tumor genes across all tumor samples. Samples are denoted in columns and genes are denoted in rows. The heatmap clearly shows that those set of genes were significantly up- or down-regulated in tumor samples from very young women. The expression level of each gene across the samples is scaled to [-3, 3] interval. These mapped expression levels are depicted using a color scale as shown at the top of the figure, as such highly expressed genes are indicated in red, intermediate in black, and weakly expressed in green. (B) Validation of microarray data by realtime RT-PCR. Ratio of expression for each gene in Young (age 35 to 45) to very young (< = 35). Red bars represent microarray hybridizations, and, and blue bars represent values from qRT-PCR. (C) Gene interaction networks analysis of genes specific to very young women tumor. Green/red indicates decreased/increased mRNA expression in younger patients compared to older counterparts. The color intensity is correlated with fold change. Straight lines are for direct gene to gene interactions, dashed lines are for indirect ones.
(DOCX)

Figure S3 **I.** Comparison of the expression profile characteristics of IDC and DCIS. (a) 143 genes have significantly different levels of expression between DCIS compared to IDC. (b) Functional enrichment analysis of genes whose expression altered between DCIS and IDC. (c-d) The network analysis of 143 genes. Green/red indicates decreased/increased mRNA expression in IDC compared to normal controls. **II.** Network analyses of genes specific to DCIS or IDC in young women (A) Venn diagram illustrating 27 genes and 94 probes (corresponding to 72 genes) that are specific to IDC and DCIS, respectively. (B) Network analyses of genes specific to IDC. Green/red indicates decreased/increased mRNA expression in IDC compared to normal controls. (C) Network analyses of genes specific to DCIS (top two significant networks shown). Green/red indicates decreased/increased mRNA expression in DCIS compared to normal controls. The color intensity is correlated with fold change. Straight lines are for direct gene to gene interactions, dashed lines are for indirect ones. DCIS: ductal carcinoma in situ; IDC: invasive ductal carcinoma.
(PDF)

Figure S4 ***In Silico*** **Independent Validation Analysis.** (A) Re-analyzed dataset from Miller et al [54] that was composed of 251 human tumor samples, of which 31 were derived from young women, which was used in the re-analysis. Our progression signature gene list was sufficient to separate patients in Miller *et. al*.’s study into two clusters which differed significantly with the p53 mutation status. The cluster which had high expression of these genes comprised nearly of all the p53 mutant tumors. (B) GSE7390 [52] and GSE12093 [53] datasets were used for independent validation analyses. Genes, including RRM2, *BIRC5, TOP2A*, *NUSAP1,* TPX2, and *CCNB2* were of significant clinical relevance for identifying patients at high risk patients groups (result for *RRM2* has been shown).
(DOCX)

Table S1 **Gene signatures specific to malignant stage of invasive ductal carcinoma (IDC) and pre-invasive ductal carcinoma** ***in situ*** **(DCIS) in young women.**
(DOCX)
