# Supplementary material for: Age-Specific Gene Expression Signatures for Breast Tumors and Cross-Species Conserved Potential Cancer Progression Markers in Young Women
Source: PLoS One. 2013 May 21;8(5):e63204. doi: 10.1371/journal.pone.0063204 (PMC3660335; doi:10.1371/journal.pone.0063204)

**Figure S1 (A)** Comparison of each age cohort, young women (≤ 45 years), 45 to 55 years (pre) and ≥ 55 years (post), with the age-matched normal controls. We identified 2632, 2029 and 2842 significantly dysregulated genes (up or down) due to tumor in young, pre and old cohorts respectively (adjusted p value < 5% and FC >2). (B) Gene interaction networks analysis of differentially expressed genes associated with tumor in each age cohort. Green/red indicates decreased/increased mRNA expression in patients compared to age-matched normal controls. The color intensity is correlated with fold change. Straight lines are for direct gene to gene interactions, dashed lines are for indirect ones (top scoring networks are shown).

(A)


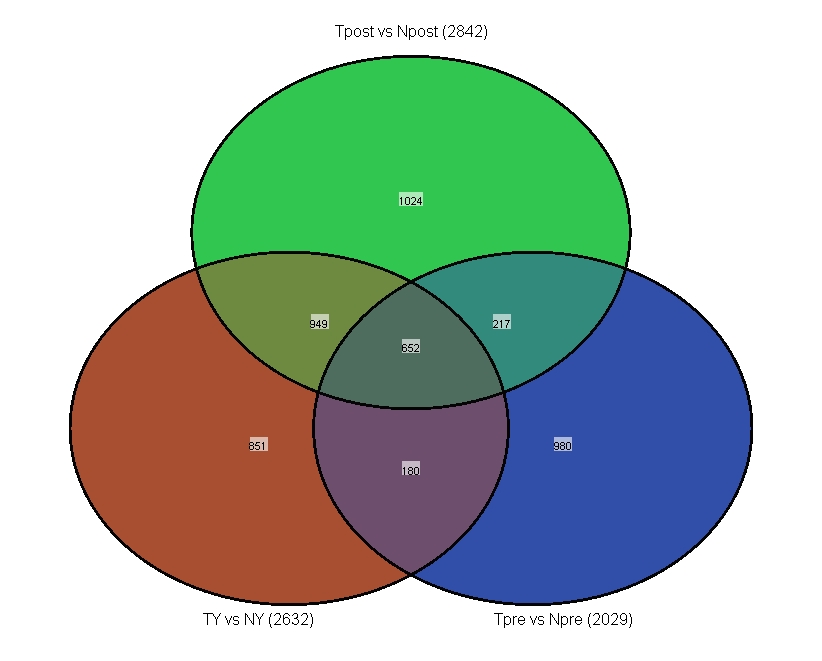


(B)

TY vs NY TPre vs NPre


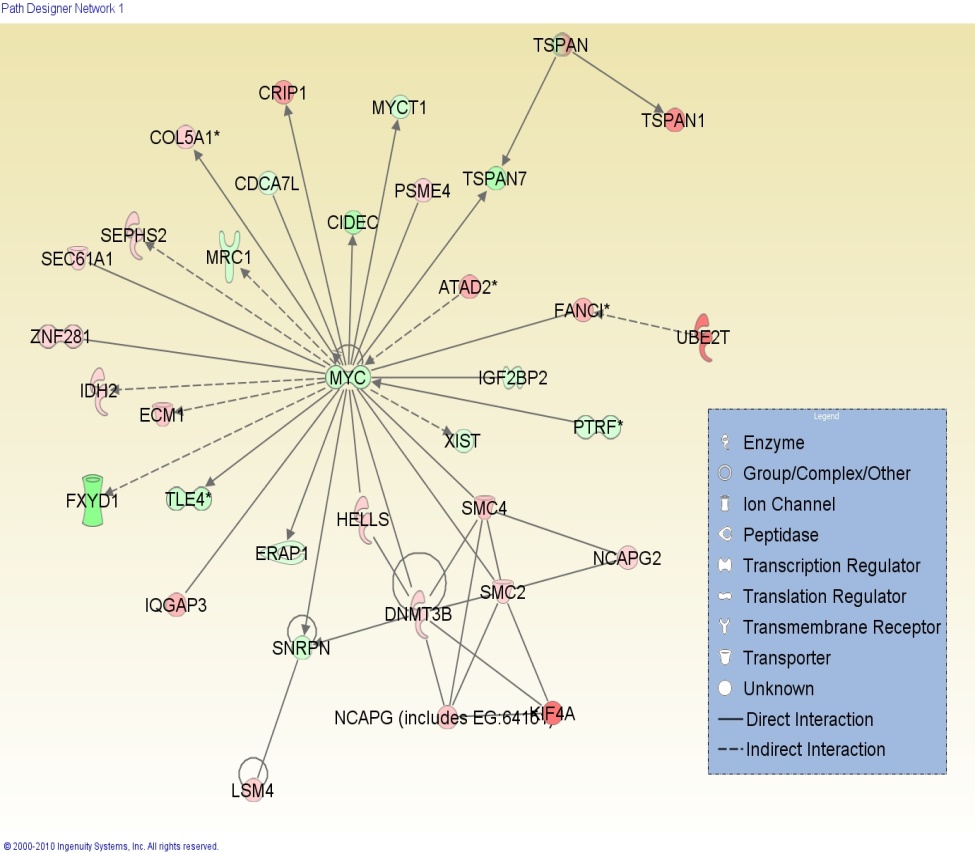

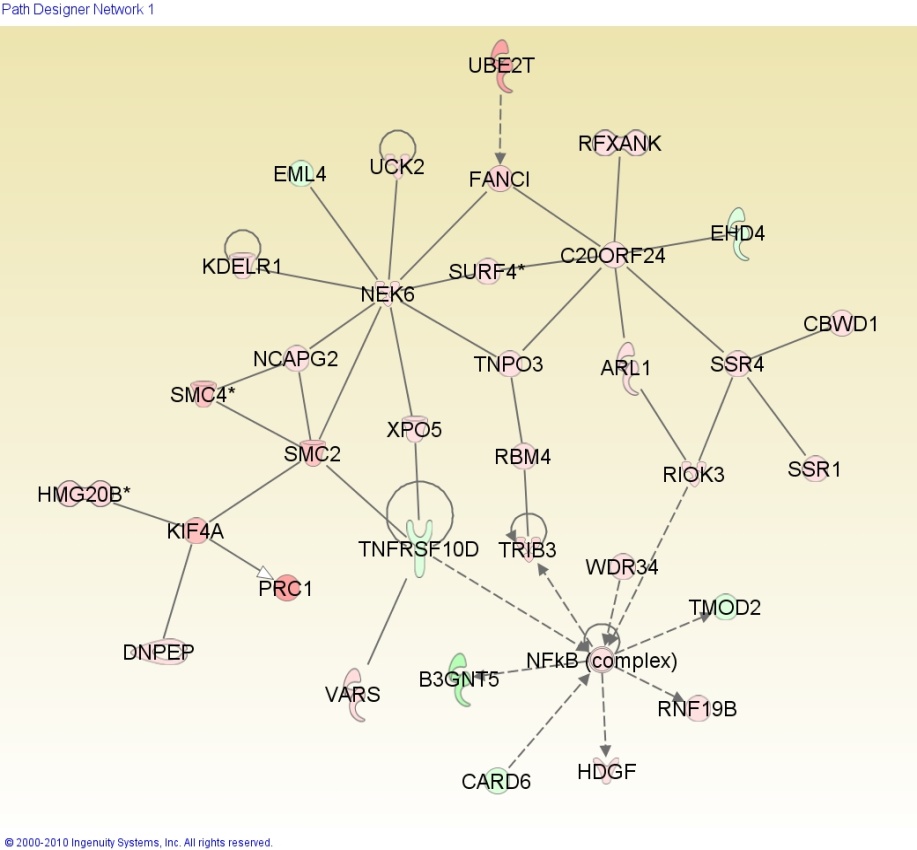


TOld vs NOld


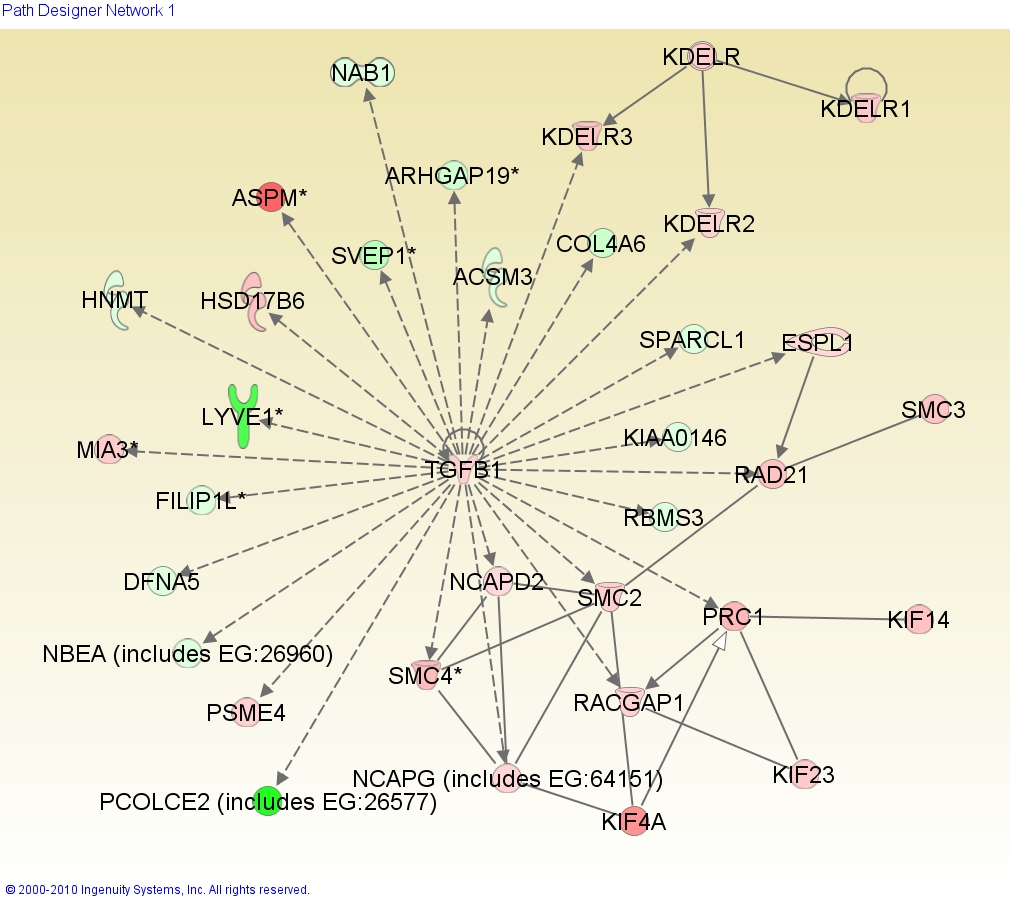

Supplement: Figure S1 — (A) Comparison of each age cohort, young women (≤45 years), 45 to 55 years (pre) and ≥55 years (post), with the age-matched normal controls. We identified 2632, 2029 and 2842 significantly dysregulated genes (up or down) due to tumor in young, pre and old cohorts respectively (adjusted p value <5% and FC >2). (B) Gene interaction networks analysis of differentially expressed genes associated with tumor in each age cohort. Green/red indicates decreased/increased mRNA expression in patients compared to age-matched normal controls. The color intensity is correlated with fold change. Straight lines are for direct gene to gene interactions, dashed lines are for indirect ones (top scoring networks are shown). (DOCX) [file pone.0063204.s001.docx]
