# Supplementary material for: Age-Specific Gene Expression Signatures for Breast Tumors and Cross-Species Conserved Potential Cancer Progression Markers in Young Women
Source: PLoS One. 2013 May 21;8(5):e63204. doi: 10.1371/journal.pone.0063204 (PMC3660335; doi:10.1371/journal.pone.0063204)

**Figure S2. (A)** Heatmap of very young-specific tumor genes across all tumor samples. Samples are denoted in columns and genes are denoted in rows. The heatmap clearly shows that those set of genes were significantly up- or down-regulated in tumor samples from very young women. The expression level of each gene across the samples is scaled to [-3, 3] interval. These mapped expression levels are depicted using a color scale as shown at the top of the figure, as such highly expressed genes are indicated in red, intermediate in black, and weakly expressed in green. (B) Validation of microarray data by realtime RT-PCR. Ratio of expression for each gene in Young (age 35 to 45) to very young (<=35). Red bars represent microarray hybridizations, and, and blue bars represent values from qRT-PCR. (C) Gene interaction networks analysis of genes specific to very young women tumor. Green/red indicates decreased/increased mRNA expression in younger patients compared to older counterparts. The color intensity is correlated with fold change. Straight lines are for direct gene to gene interactions, dashed lines are for indirect ones.

**(A)**

**
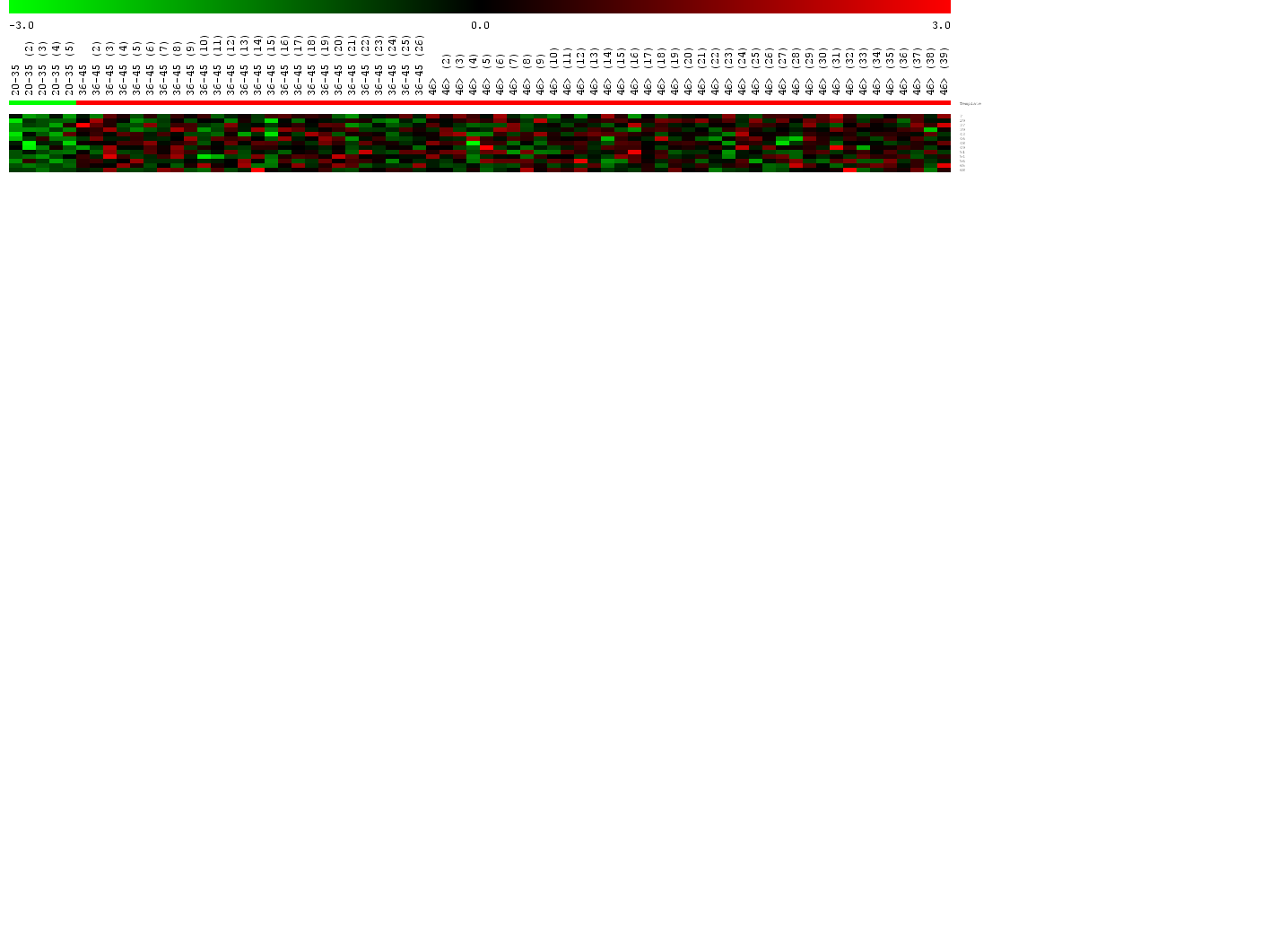

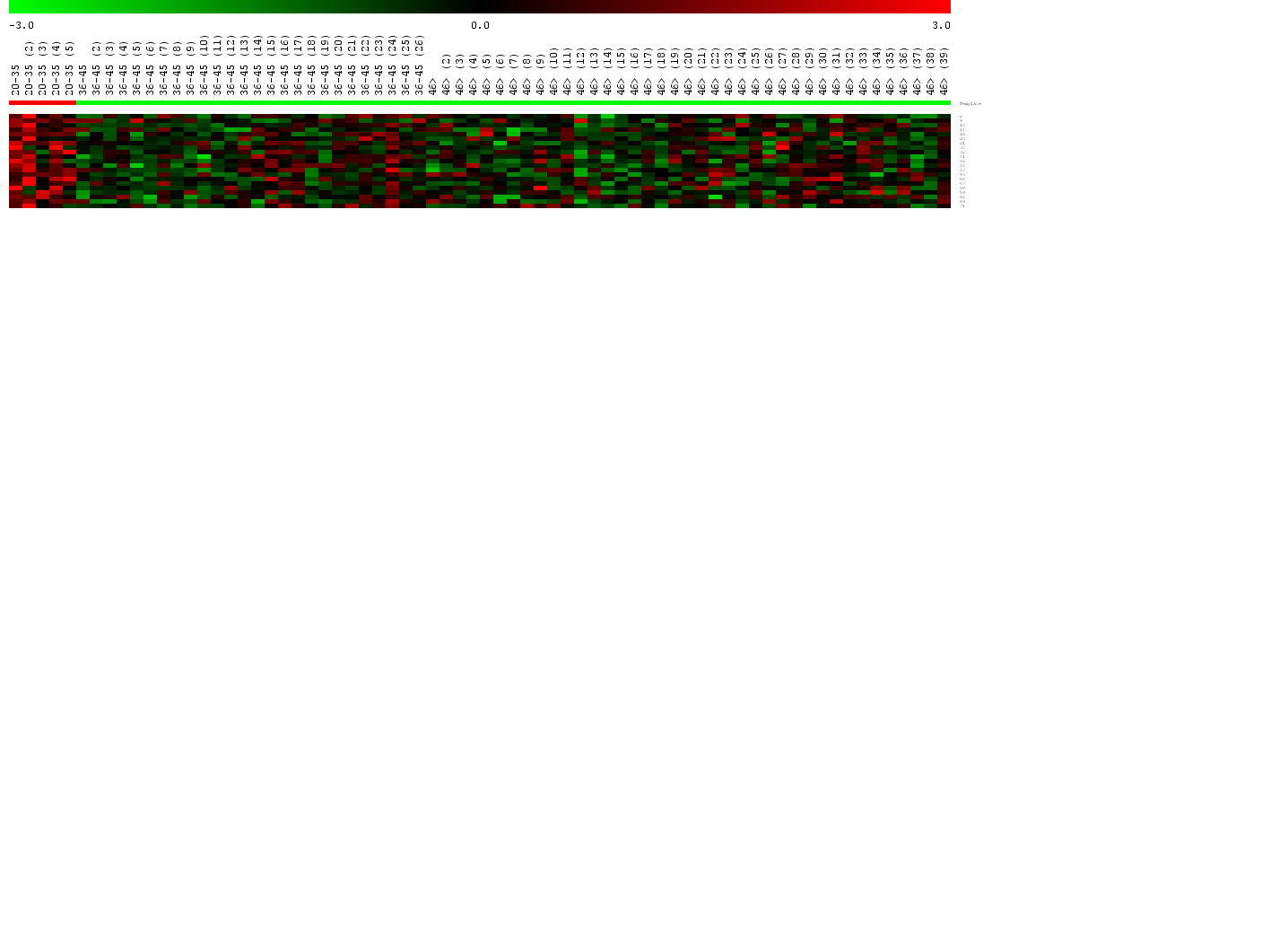
**

**(B)**

(C)


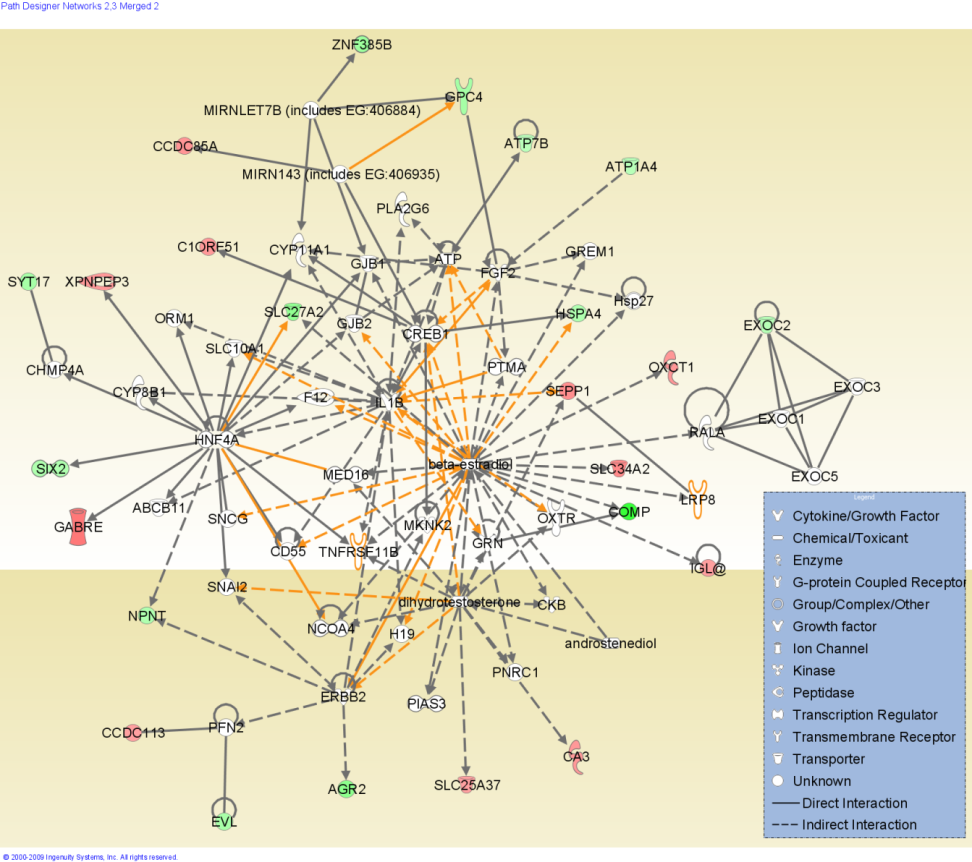

Supplement: Figure S2 — (A) Heatmap of very young-specific tumor genes across all tumor samples. Samples are denoted in columns and genes are denoted in rows. The heatmap clearly shows that those set of genes were significantly up- or down-regulated in tumor samples from very young women. The expression level of each gene across the samples is scaled to [-3, 3] interval. These mapped expression levels are depicted using a color scale as shown at the top of the figure, as such highly expressed genes are indicated in red, intermediate in black, and weakly expressed in green. (B) Validation of microarray data by realtime RT-PCR. Ratio of expression for each gene in Young (age 35 to 45) to very young (< = 35). Red bars represent microarray hybridizations, and, and blue bars represent values from qRT-PCR. (C) Gene interaction networks analysis of genes specific to very young women tumor. Green/red indicates decreased/increased mRNA expression in younger patients compared to older counterparts. The color intensity is correlated with fold change. Straight lines are for direct gene to gene interactions, dashed lines are for indirect ones. (DOCX) [file pone.0063204.s002.docx]
