# Supplementary material for: Age-Specific Gene Expression Signatures for Breast Tumors and Cross-Species Conserved Potential Cancer Progression Markers in Young Women
Source: PLoS One. 2013 May 21;8(5):e63204. doi: 10.1371/journal.pone.0063204 (PMC3660335; doi:10.1371/journal.pone.0063204)
Supplement: Figure S3 — I. Comparison of the expression profile characteristics of IDC and DCIS. (a) 143 genes have significantly different levels of expression between DCIS compared to IDC. (b) Functional enrichment analysis of genes whose expression altered between DCIS and IDC. (c-d) The network analysis of 143 genes. Green/red indicates decreased/increased mRNA expression in IDC compared to normal controls. II. Network analyses of genes specific to DCIS or IDC in young women (A) Venn diagram illustrating 27 genes and 94 probes (corresponding to 72 genes) that are specific to IDC and DCIS, respectively. (B) Network analyses of genes specific to IDC. Green/red indicates decreased/increased mRNA expression in IDC compared to normal controls. (C) Network analyses of genes specific to DCIS (top two significant networks shown). Green/red indicates decreased/increased mRNA expression in DCIS compared to normal controls. The color intensity is correlated with fold change. Straight lines are for direct gene to gene interactions, dashed lines are for indirect ones. DCIS: ductal carcinoma in situ; IDC: invasive ductal carcinoma. (PDF) [file pone.0063204.s003.pdf]

Figure S3. I. Comparison of the expression profile characteristics of IDC and DCIS. (a) 143 genes have significantly different levels of expression between DCIS compared to IDC. (b) Functional enrichment analysis of genes whose expression altered between DCIS and IDC. (c-d) The network analysis of 143 genes. Green/red indicates decreased/increased mRNA expression in IDC compared to normal controls.

II. Network analyses of genes specific to DCIS or IDC in young women (A) Venn diagram illustrating 27 genes and 94 probes (corresponding to 72 genes) that are specific to IDC and DCIS, respectively. (B) Network analyses of genes specific to IDC. Green/red indicates decreased/increased mRNA expression in IDC compared to normal controls. (C) Network analyses of genes specific to DCIS (top two significant networks shown). Green/red indicates decreased/increased mRNA expression in DCIS compared to normal controls. The color intensity is correlated with fold change. Straight lines are for direct gene to gene interactions, dashed lines are for indirect ones. DCIS: ductal carcinoma in situ; IDC: invasive ductal carcinoma.

(I)

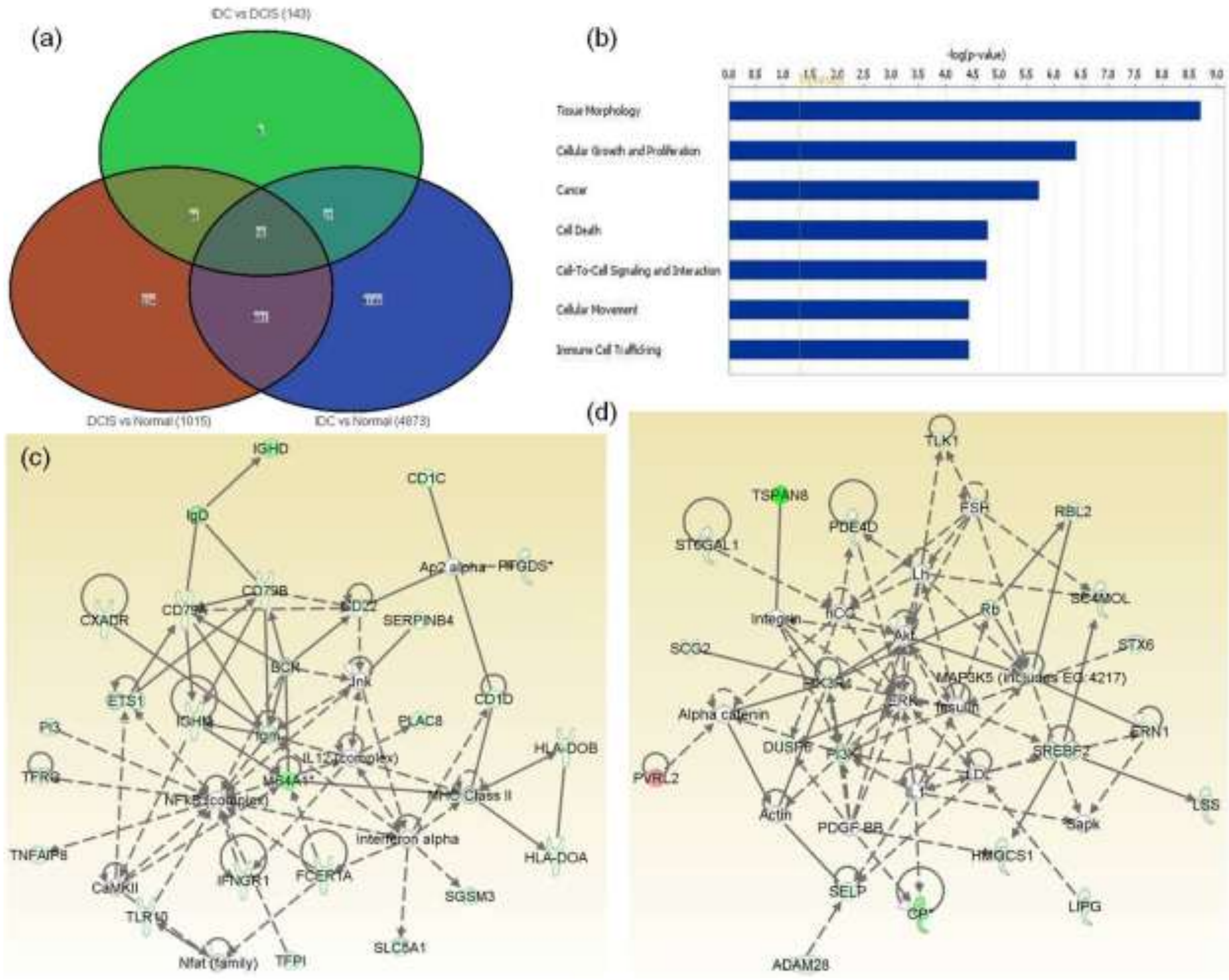

(a)
IDC vs DCIS (143)
DCIS vs Normal (1015)
IDC vs Normal (4873)
(b)
-log(p-value)
0.0
0.5
1.0
1.5
2.0
2.5
3.0
3.5
4.0
4.5
5.0
5.5
6.0
6.5
7.0
7.5
8.0
8.5
9.0
Tissue Morphology
Cellular Growth and Proliferation
Cancer
Cell Death
Cell-To-Cell Signaling and Interaction
Cellular Movement
Immune Cell Trafficking
(c)
IGHD
CD1C
IgD
Ap2 alpha
PTGDS*
CD79B
CXADR
CD79A
CD22
SERPINB4
BCR
Jnk
ETS1
CD1D
PI3
IGHM
PLAC8
IL12 (complex)
HLA-DOB
TFRC
MS4A1*
NFkB (complex)
MHC Class II
Interferon alpha
TNFAIP8
IFNGR1
FCER1A
HLA-DOA
CaMKII
SGSM3
TLR10
Nfat (family)
TFPI
SLC5A1
(d)
TLK1
TSPAN8
FSH
RBL2
ST6GAL1
PDE4D
Lh
SC4MOL
Integrin
Rb
Akt
STX6
SCG2
MAP3K5 (includes EG:4217)
ERK
Insulin
Alpha catenin
ERN1
DUSP6
PI3K
SREBF2
PVRL2
LDL
LSS
Actin
PDGF BB
Sapk
HMGCS1
SELP
CP*
LIPG
ADAM28

(II)

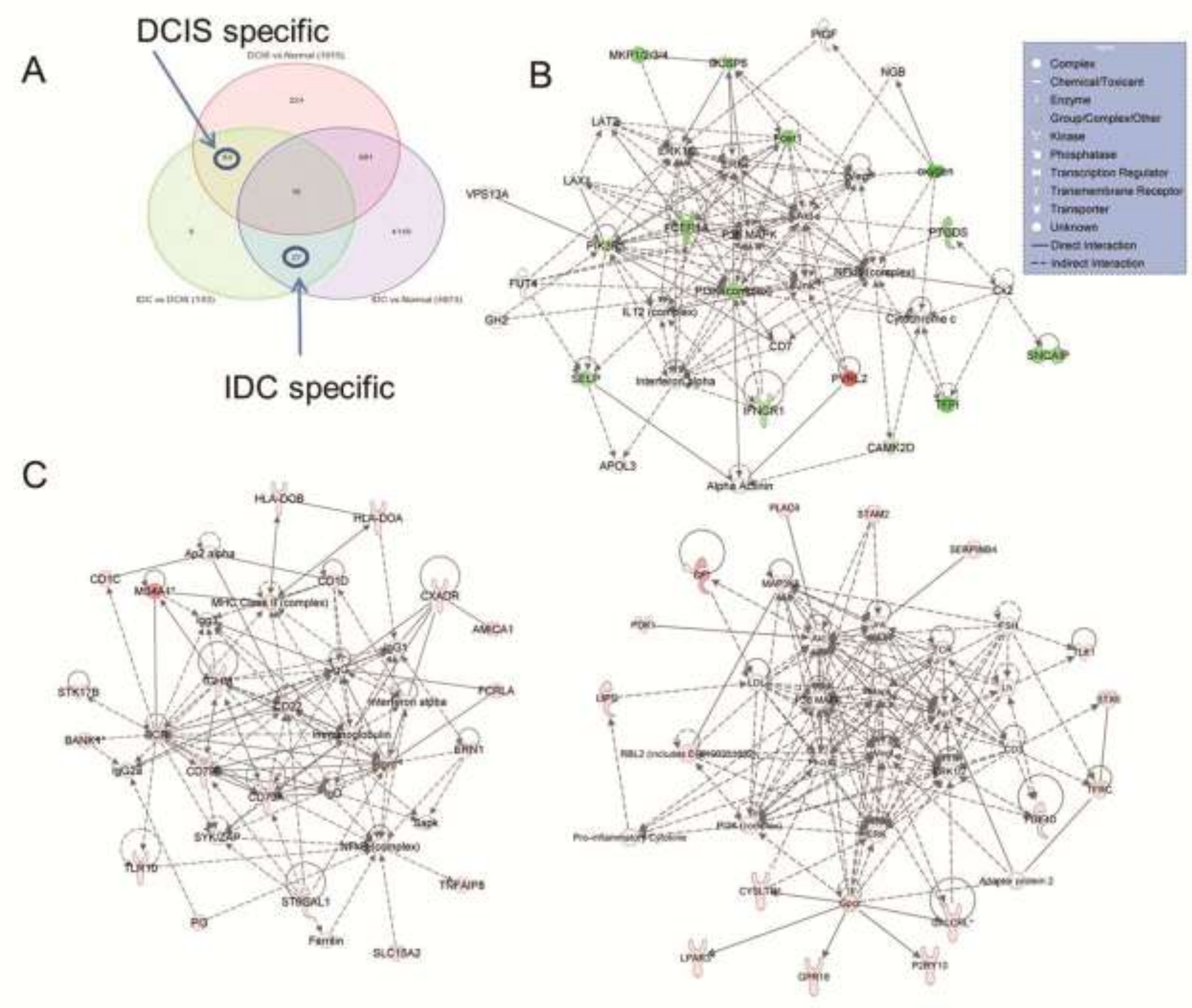

A
DCIS specific
IDC specific
B
PIGF
MKR1/2/3/4
NGB
LAT2
LAX1
VPS13A
FUT4
GH2
IL12 (complex)
CD7
Cytochrome c
SELP
Interferon alpha
PVRL2
IFNGR1
TFR
SNCAIP
PTGDS
CAMK2D
APOL3
Alpha Actinin
NFkB (complex)
P38 MAPK
Complex
Chemical/Toxicant
Enzyme
Group/Complex/Other
Kinase
Phosphatase
Transcription Regulator
Transmembrane Receptor
Transporter
Unknown
Direct Interaction
Indirect Interaction
C
HLA-DOB
HLA-DOA
Ap2 alpha
CD1C
CD1D
MS4A1
MHC Class II (complex)
CXADR
AMICA1
STK17B
FCRLA
Interferon alpha
BANK1
Immunoglobulin
ERN1
IgG2b
CD79B
CD79A
SYK/ZAP
NFkB (complex)
TLR10
ST6GAL1
TNFAIP8
PIGF
Ferritin
SLC15A2
Pro-inflammatory Cytokine
P38 MAPK
Adaptor protein 2
