# Supplementary material for: Age-Specific Gene Expression Signatures for Breast Tumors and Cross-Species Conserved Potential Cancer Progression Markers in Young Women
Source: PLoS One. 2013 May 21;8(5):e63204. doi: 10.1371/journal.pone.0063204 (PMC3660335; doi:10.1371/journal.pone.0063204)
Supplement: Figure S4 — In Silico Independent Validation Analysis. (A) Re-analyzed dataset from Miller et al [54] that was composed of 251 human tumor samples, of which 31 were derived from young women, which was used in the re-analysis. Our progression signature gene list was sufficient to separate patients in Miller et. al.’s study into two clusters which differed significantly with the p53 mutation status. The cluster which had high expression of these genes comprised nearly of all the p53 mutant tumors. (B) GSE7390 [52] and GSE12093 [53] datasets were used for independent validation analyses. Genes, including RRM2, BIRC5, TOP2A, NUSAP1, TPX2, and CCNB2 were of significant clinical relevance for identifying patients at high risk patients groups (result for RRM2 has been shown). (DOCX) [file pone.0063204.s004.docx]

**Figure S4.** *in silico* Independent Validation Analysis. (A) The dataset from Miller et al [1] was composed of 251 human tumor samples, of which 31 were derived from young women, which we used in our re-analysis. Our progression signature gene list was sufficient to separate patients in Miller *et. al*.'s study into two clusters which differed significantly with the p53 mutation status. The cluster which had high expression of these genes comprised nearly of all the p53 mutant tumors. (B) GSE7390 [2] and GSE12093 [3] datasets were used for independent validation analyses. Genes, including *RRM2, BIRC5, TOP2A, NUSAP1, TPX2, and CCNB2* were of significant clinical relevance for identifying patients at high risk patients groups (result for *RRM2* has been shown)

**(A)**


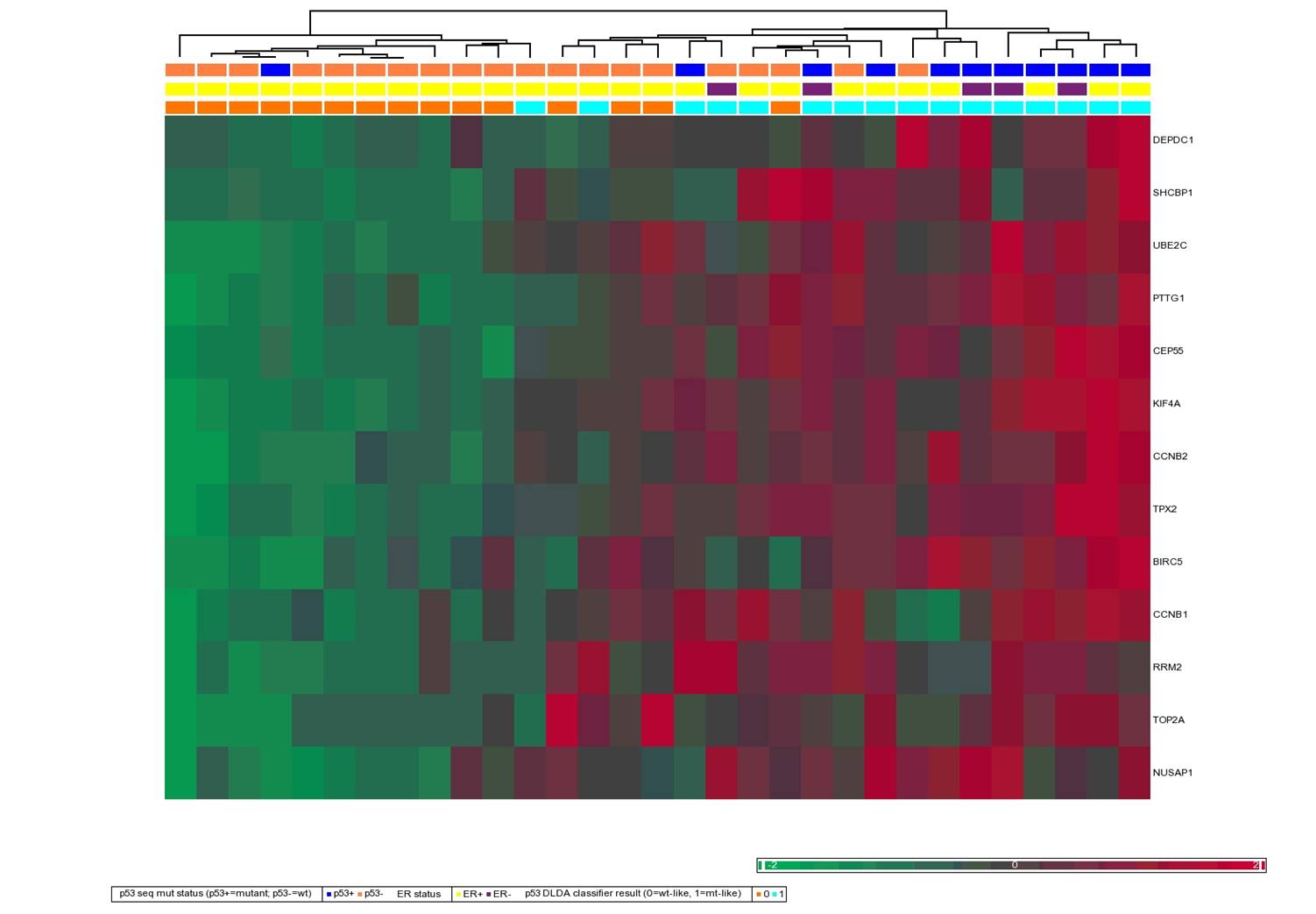


**(B)**


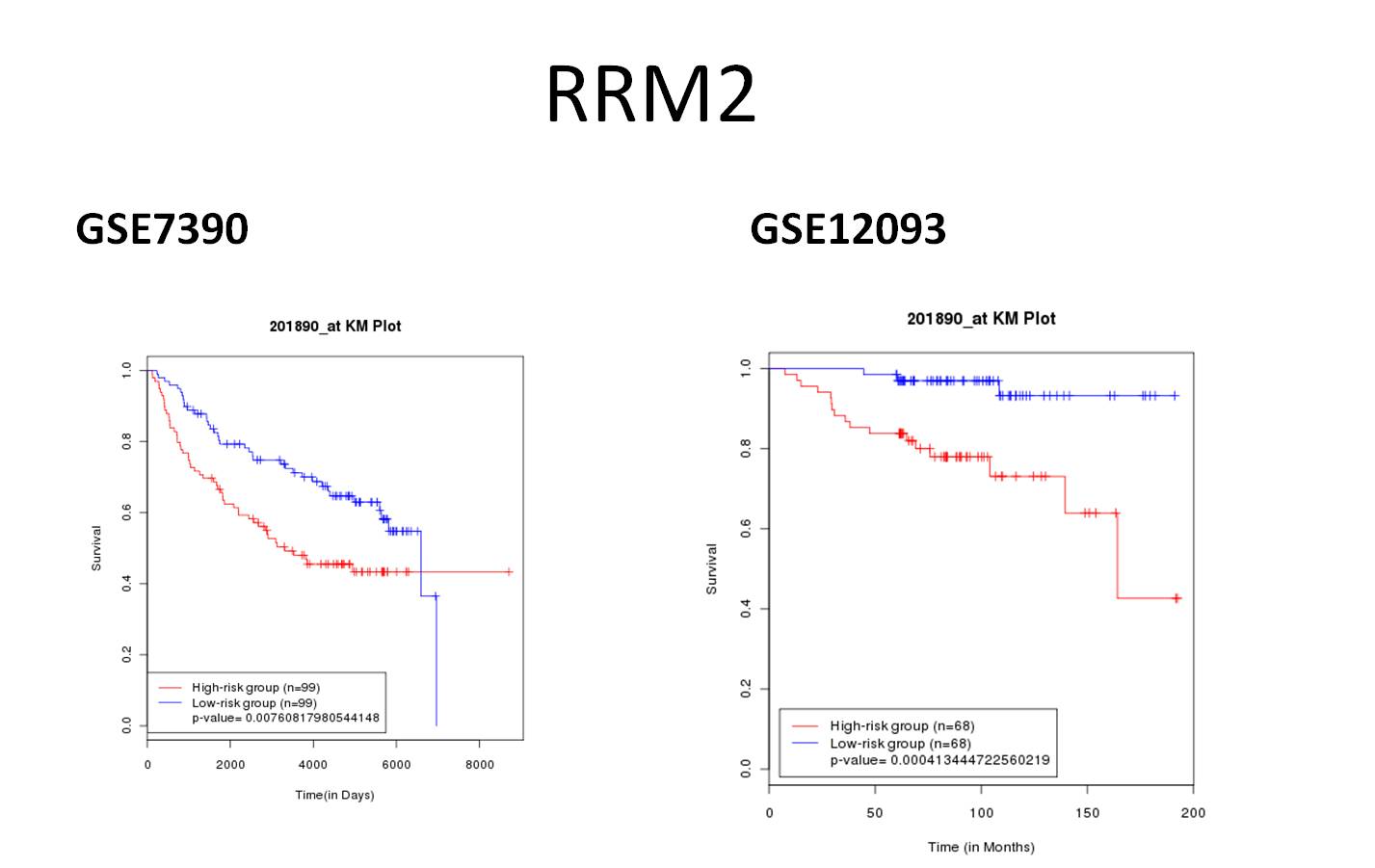


1. Miller LD, Smeds J, George J, Vega VB, Vergara L, et al. (2005) An expression signature for p53 status in human breast cancer predicts mutation status, transcriptional effects, and patient survival. Proc Natl Acad Sci U S A 102: 13550-13555.

2. Desmedt C, Piette F, Loi S, Wang Y, Lallemand F, et al. (2007) Strong time dependence of the 76-gene prognostic signature for node-negative breast cancer patients in the TRANSBIG multicenter independent validation series. Clin Cancer Res 13: 3207-3214.

3. Zhang Y, Sieuwerts AM, McGreevy M, Casey G, Cufer T, et al. (2009) The 76-gene signature defines high-risk patients that benefit from adjuvant tamoxifen therapy. Breast Cancer Res Treat 116: 303-309.
