# Supplementary material for: Age-Specific Gene Expression Signatures for Breast Tumors and Cross-Species Conserved Potential Cancer Progression Markers in Young Women
Source: PLoS One. 2013 May 21;8(5):e63204. doi: 10.1371/journal.pone.0063204 (PMC3660335; doi:10.1371/journal.pone.0063204)
Supplement: Table S1 — Gene signatures specific to malignant stage of invasive ductal carcinoma (IDC) and pre-invasive ductal carcinoma in situ (DCIS) in young women. (DOCX) [file pone.0063204.s005.docx]

**Table S1.** Gene signatures specific to malignant stage of invasive ductal carcinoma (IDC) and pre-invasive ductal carcinoma *in situ* (DCIS) in young women.

**a) IDC specific genes:**

| Gene Symbol | Gene Title | p-value  (DCIS vs N) | FC  (DCIS vsN) | p-value  (IDC vs N) | FC  (IDC vs N) |
| --- | --- | --- | --- | --- | --- |
| CACNB3 | calcium channel, voltage-dependent, beta 3 subunit | 0.573824 | -1.17 | 2.79E-08 | 2.21 |
| ZNF239 | zinc finger protein 239 | 0.026662 | -2.72 | 0.012008 | 1.73 |
| TMEM160 | transmembrane protein 160 | 0.240569 | -1.31 | 5.32E-06 | 1.69 |
| PVRL2 | poliovirus receptor-related 2 (herpesvirus entry mediator B) | 0.252318 | -1.28 | 5.61E-06 | 1.63 |
| RGS18 | regulator of G-protein signaling 18 | 0.011594 | 2.65 | 0.013916 | -1.58 |
| GUCY1A3 | Guanylate cyclase 1, soluble, alpha 3 | 0.015662 | 2.62 | 0.016999 | -1.58 |
| CAMK2D | Calcium/calmodulin-dependent protein kinase (CaM kinase) II delta | 0.028484 | 2.23 | 0.006545 | -1.62 |
| KIAA0746 | KIAA0746 protein /// serine incorporator 2 | 0.013087 | 3.07 | 0.024285 | -1.63 |
| IFNGR1 | interferon gamma receptor 1 | 0.033126 | 2.15 | 0.002295 | -1.70 |
| ATP13A4 | ATPase type 13A4 | 0.010114 | 3.21 | 0.012502 | -1.72 |
| LRMP | lymphoid-restricted membrane protein | 0.046896 | 2.14 | 0.001952 | -1.78 |
| SELP | selectin P (granule membrane protein 140kDa, antigen CD62) | 0.067217 | 2.01 | 0.001592 | -1.80 |
| FAM65B | family with sequence similarity 65, member B | 0.034515 | 2.71 | 0.005868 | -1.88 |
| PTGDS | prostaglandin D2 synthase 21kDa (brain) | 0.029932 | 2.85 | 0.003959 | -1.96 |
| LRMP | lymphoid-restricted membrane protein | 0.087289 | 1.98 | 0.000456 | -2.00 |
| PIK3R1 | phosphoinositide-3-kinase, regulatory subunit 1 (alpha) | 0.076099 | 2.19 | 0.001185 | -2.02 |
| SNCAIP | synuclein, alpha interacting protein | 0.059184 | 1.86 | 2.24E-06 | -2.19 |
| PTGDS | prostaglandin D2 synthase 21kDa (brain) | 0.032049 | 3.43 | 0.003686 | -2.25 |
| ATP8A1 | ATPase, aminophospholipid transporter (APLT), class I, type 8A, member 1 | 0.285663 | 1.48 | 8.24E-06 | -2.27 |
| DUSP6 | dual specificity phosphatase 6 | 0.273132 | 1.57 | 9.27E-06 | -2.51 |
| IGHD | immunoglobulin heavy constant delta | 0.012835 | 9.60 | 0.032059 | -2.54 |
| FCER1A | Fc fragment of IgE, high affinity I, receptor for; alpha polypeptide | 0.041447 | 4.41 | 0.006927 | -2.59 |
| PTGDS | prostaglandin D2 synthase 21kDa (brain) | 0.042272 | 3.86 | 0.00285 | -2.62 |
| TFPI | tissue factor pathway inhibitor (lipoprotein-associated coagulation inhibitor) | 0.172106 | 1.83 | 5.37E-08 | -3.45 |
| P2RY14 | purinergic receptor P2Y, G-protein coupled, 14 | 0.360726 | 1.51 | 6.83E-08 | -3.49 |
| C7 | complement component 7 | 0.123732 | 2.89 | 3.81E-05 | -4.12 |
| HSD17B2 | hydroxysteroid (17-beta) dehydrogenase 2 | 0.118183 | 3.11 | 4.67E-05 | -4.36 |

**b) DCIS specific genes:**

| Gene Symbol | Gene Title | p-value  (DCIS vs N) | FC  (DCIS vsN) | p-value (IDC vs N) | FC (IDC vs N) |
| --- | --- | --- | --- | --- | --- |
| TSPAN8 | tetraspanin 8 | 2.32E-05 | 61.72 | 0.822639 | 1.11 |
| ABCC11 | ATP-binding cassette, sub-family C (CFTR/MRP), member 11 | 3.52E-05 | 60.64 | 0.071022 | 2.29 |
| MS4A1 | membrane-spanning 4-domains, subfamily A, member 1 | 3.93E-05 | 32.83 | 0.729048 | 1.14 |
| CP | ceruloplasmin (ferroxidase) /// similar to Ceruloplasmin precursor (Ferroxidase) | 2.05E-06 | 25.02 | 0.976303 | -1.01 |
| TMC5 | transmembrane channel-like 5 | 6.47E-07 | 20.07 | 0.059039 | 1.68 |
| CP | ceruloplasmin (ferroxidase) | 0.000939 | 20.01 | 0.271762 | -1.59 |
| PEG10 | paternally expressed 10 | 0.002221 | 19.11 | 0.209625 | -1.77 |
| CD1E | CD1e molecule | 2.56E-08 | 14.46 | 0.942819 | 1.02 |
| PLAC8 | placenta-specific 8 | 0.000377 | 10.07 | 0.503202 | -1.22 |
| KMO | kynurenine 3-monooxygenase (kynurenine 3-hydroxylase) | 3.52E-08 | 8.40 | 0.094557 | 1.33 |
| CD1C | CD1c molecule | 4.58E-05 | 7.68 | 0.087225 | -1.49 |
| IGLV4-60 | immunoglobulin lambda variable 4-60 | 0.001238 | 7.24 | 0.281041 | -1.36 |
| SCG2 | secretogranin II (chromogranin C) | 4.48E-06 | 7.16 | 0.121188 | 1.36 |
| TFRC | transferrin receptor (p90, CD71) | 2.48E-05 | 6.98 | 0.07559 | 1.46 |
| BANK1 | B-cell scaffold protein with ankyrin repeats 1 | 0.000706 | 6.41 | 0.364747 | -1.26 |
| IGHM | immunoglobulin heavy constant mu | 0.000443 | 6.06 | 0.365994 | -1.24 |
| P2RY10 | purinergic receptor P2Y, G-protein coupled, 10 | 5.64E-05 | 5.85 | 0.076645 | 1.43 |
| PI3 | peptidase inhibitor 3, skin-derived (SKALP) | 0.004049 | 5.43 | 0.055108 | -1.71 |
| HLA-DOB | major histocompatibility complex, class II, DO beta | 0.001031 | 5.32 | 0.522031 | -1.16 |
| MYO19 | myosin XIX | 9.91E-07 | 5.17 | 0.036324 | 1.38 |
| CALCRL | calcitonin receptor-like | 0.000452 | 5.16 | 0.873163 | 1.04 |
| CYSLTR1 | cysteinyl leukotriene receptor 1 | 0.000109 | 5.07 | 0.645137 | 1.09 |
| AP1S3 | adaptor-related protein complex 1, sigma 3 subunit | 6.26E-07 | 4.95 | 0.024996 | 1.39 |
| UGT1A1 | UDP glucuronosyltransferase 1 family, polypeptide A1 /// UDP glucuronosyltransfe | 2.93E-12 | 4.93 | 0.130275 | 1.16 |
| GGNBP2 | gametogenetin binding protein 2 | 1.46E-05 | 4.93 | 0.536398 | 1.11 |
| SLC5A1 | solute carrier family 5 (sodium/glucose cotransporter), member 1 | 0.006006 | 4.88 | 0.057934 | -1.68 |
| CD79A | CD79a molecule, immunoglobulin-associated alpha | 0.002026 | 4.87 | 0.237712 | -1.33 |
| GPR18 | G protein-coupled receptor 18 | 0.00036 | 4.78 | 0.95669 | 1.01 |
| ACOX1 | acyl-Coenzyme A oxidase 1, palmitoyl | 2.55E-07 | 4.74 | 0.033173 | 1.34 |
| ZNHIT3 | zinc finger, HIT type 3 | 2.41E-06 | 4.73 | 0.893801 | 1.02 |
| PCDH7 | protocadherin 7 | 1.97E-05 | 4.34 | 0.144267 | 1.26 |
| STEAP4 | STEAP family member 4 | 0.000101 | 4.30 | 0.672019 | 1.08 |
| FCRL5 | Fc receptor-like 5 | 0.00245 | 4.15 | 0.168467 | -1.36 |
| GUCY1A3 | guanylate cyclase 1, soluble, alpha 3 | 0.001644 | 3.90 | 0.077862 | -1.43 |
| CXADR | coxsackie virus and adenovirus receptor | 8.37E-05 | 3.81 | 0.301382 | 1.18 |
| PDE4D | phosphodiesterase 4D, cAMP-specific (phosphodiesterase E3 dunce homolog, Drosoph | 0.000615 | 3.81 | 0.824477 | 1.04 |
| CDK3 | cyclin-dependent kinase 3 /// hypothetical protein LOC100134934 | 0.000175 | 3.70 | 0.70711 | 1.06 |
| AMICA1 | adhesion molecule, interacts with CXADR antigen 1 | 0.002928 | 3.55 | 0.272325 | -1.25 |
| LPAR3 | lysophosphatidic acid receptor 3 | 9.37E-06 | 3.46 | 0.419588 | 1.11 |
| SGSM3 | small G protein signaling modulator 3 | 9.63E-08 | 3.27 | 0.601223 | 1.05 |
| LIPG | lipase, endothelial | 4.40E-05 | 3.26 | 0.667861 | 1.06 |
| TLK1 | tousled-like kinase 1 | 0.000171 | 3.25 | 0.584826 | 1.08 |
| RBL2 | retinoblastoma-like 2 (p130) | 0.000856 | 3.23 | 0.955889 | -1.01 |
| HMGCS1 | 3-hydroxy-3-methylglutaryl-Coenzyme A synthase 1 (soluble) | 3.64E-06 | 3.19 | 0.002558 | 1.42 |
| SREBF2 | sterol regulatory element binding transcription factor 2 | 1.83E-06 | 3.09 | 0.049346 | 1.24 |
| ST6GAL1 | ST6 beta-galactosamide alpha-2,6-sialyltranferase 1 | 0.004826 | 3.07 | 0.190278 | -1.28 |
| ERN1 | endoplasmic reticulum to nucleus signaling 1 | 0.003566 | 3.07 | 0.136357 | -1.31 |
| CACNA2D3 | calcium channel, voltage-dependent, alpha 2/delta subunit 3 | 1.29E-06 | 2.99 | 0.782991 | 1.03 |
| FAM65B | family with sequence similarity 65, member B | 0.001307 | 2.94 | 0.176399 | -1.24 |
| TLR10 | toll-like receptor 10 | 1.11E-07 | 2.91 | 0.411698 | -1.08 |
| ST3GAL5 | ST3 beta-galactoside alpha-2,3-sialyltransferase 5 | 0.001724 | 2.86 | 0.270495 | -1.19 |
| DNAJC3 | DnaJ (Hsp40) homolog, subfamily C, member 3 | 0.000158 | 2.86 | 0.334931 | 1.13 |
| CD1D | CD1d molecule | 0.000874 | 2.86 | 0.647678 | -1.07 |
| TNFAIP8 | tumor necrosis factor, alpha-induced protein 8 | 0.004119 | 2.83 | 0.10457 | -1.32 |
| FCRLA | Fc receptor-like A | 0.00082 | 2.78 | 0.252867 | -1.18 |
| PDK1 | pyruvate dehydrogenase kinase, isozyme 1 | 0.000212 | 2.73 | 0.607421 | 1.07 |
| TMEM132A | transmembrane protein 132A | 1.42E-09 | 2.72 | 0.000339 | 1.31 |
| CD79B | CD79b molecule, immunoglobulin-associated beta | 0.006284 | 2.69 | 0.082864 | -1.35 |
| MAP3K5 | mitogen-activated protein kinase kinase kinase 5 | 0.000899 | 2.68 | 0.048481 | -1.32 |
| NUP50 | nucleoporin 50kDa | 2.38E-05 | 2.61 | 0.060951 | 1.22 |
| STX6 | syntaxin 6 | 3.53E-08 | 2.57 | 3.93E-05 | 1.39 |
| ADAM28 | ADAM metallopeptidase domain 28 | 0.001992 | 2.46 | 0.501061 | -1.10 |
| LSS | lanosterol synthase (2,3-oxidosqualene-lanosterol cyclase) | 8.40E-07 | 2.43 | 0.070021 | 1.16 |
| SERPINB3 | serpin peptidase inhibitor, clade B (ovalbumin), member 3 /// serpin peptidase i | 0.000651 | 2.34 | 0.850093 | 1.02 |
| TMEM86A | transmembrane protein 86A | 2.94E-05 | 2.14 | 0.499888 | 1.06 |
| TRAF3IP3 | hypothetical protein LOC100133233 /// TRAF3 interacting protein 3 | 1.88E-05 | 2.14 | 0.361685 | -1.08 |
| HLA-DOA | major histocompatibility complex, class II, DO alpha | 0.000122 | 2.14 | 0.622278 | 1.05 |
| STAM2 | signal transducing adaptor molecule (SH3 domain and ITAM motif) 2 | 8.34E-09 | 2.12 | 0.000185 | 1.25 |
| SLC26A4 | solute carrier family 26, member 4 | 0.000103 | 2.05 | 0.284198 | -1.10 |
| CD22 | CD22 molecule | 0.000116 | 2.04 | 0.208773 | 1.11 |
| WDR60 | WD repeat domain 60 | 0.001311 | -2.03 | 0.662416 | 1.05 |
| ASB13 | ankyrin repeat and SOCS box-containing 13 | 0.006099 | -2.42 | 0.250002 | 1.19 |
